# Clustering scRNA-seq data with the cross-view collaborative information fusion strategy

**DOI:** 10.1093/bib/bbae511

**Published:** 2024-10-14

**Authors:** Zhengzheng Lou, Xiaojiao Wei, Yuanhao Hu, Shizhe Hu, Yucong Wu, Zhen Tian

**Affiliations:** School of Computer and Artificial Intelligence, Zhengzhou University, Zhengzhou 450000, China; School of Computer and Artificial Intelligence, Zhengzhou University, Zhengzhou 450000, China; School of Computer and Artificial Intelligence, Zhengzhou University, Zhengzhou 450000, China; School of Computer and Artificial Intelligence, Zhengzhou University, Zhengzhou 450000, China; School of Computer and Artificial Intelligence, Zhengzhou University, Zhengzhou 450000, China; School of Computer and Artificial Intelligence, Zhengzhou University, Zhengzhou 450000, China; Yangtze Delta Region Institute (Quzhou), University of Electronic Science and Technology of China, Quzhou 324000, China

**Keywords:** scRNA-seq data clustering, cross-view, information bottleneck

## Abstract

Single-cell RNA sequencing (scRNA-seq) technology has revolutionized biological research by enabling high-throughput, cellular-resolution gene expression profiling. A critical step in scRNA-seq data analysis is cell clustering, which supports downstream analyses. However, the high-dimensional and sparse nature of scRNA-seq data poses significant challenges to existing clustering methods. Furthermore, integrating gene expression information with potential cell structure data remains largely unexplored. Here, we present scCFIB, a novel information bottleneck (IB)-based clustering algorithm that leverages the power of IB for efficient processing of high-dimensional sparse data and incorporates a cross-view fusion strategy to achieve robust cell clustering. scCFIB constructs a multi-feature space by establishing two distinct views from the original features. We then formulate the cell clustering problem as a target loss function within the IB framework, employing a collaborative information fusion strategy. To further optimize scCFIB’s performance, we introduce a novel sequential optimization approach through an iterative process. Benchmarking against established methods on diverse scRNA-seq datasets demonstrates that scCFIB achieves superior performance in scRNA-seq data clustering tasks. Availability: the source code is publicly available on GitHub: https://github.com/weixiaojiao/scCFIB.

## Introduction

Cells are regarded as the basic units of living organisms. While completing their life cycle, cells undergo molecular biology or genetic alterations that give rise to cellular diversity, i.e. cellular heterogeneity [1]. Single-cell RNA sequencing (scRNA-seq) could characterize multicellular organisms in more detail than batch RNA sequencing. It could analyze the gene expression status of an individual cell and study the heterogeneity of cells at a single-cell level, enabling us to observe biological systems at the resolution of individual cells[2]. Due to the rapid development of single-cell sequencing technology, researchers can understand the composition of complex tissues[3] and the connections between gene networks [4], monitor embryonic development [5], and gain biological insights[6]. The widespread application of single-cell sequencing technology in key fields has promoted the development of the analysis of scRNA-seq data and the design of computational methods.

Unsupervised clustering serves as a crucial means of analyzing scRNA-seq data, inferring the distinction of various cell types. However, due to technical and biological limitations, low capture rate and high dropout rate result in the high dimensionality, and high sparsity of single-cell sequencing data, which has become a major obstacle to clustering scRNA-seq data[7]. Thus an imperative requirement exists for computational approaches capable of effectively clustering single-cell data.

Early studies mainly concentrated on deriving low-dimensional representations of data by utilizing dimensionality reduction techniques like t-SNE [8]and PCA[9], followed by the application of traditional clustering methods to discern cell types. For example, t-SNE is a nonlinear dimensionality reduction technique that explores the structure of high-dimensional data. It defines a probability distribution between data points in high-dimensional space to represent their similarity and then creates a similar distribution in low-dimensional space. PCA is one of the most widely used data dimensionality reduction algorithms, where it maps n-dimensional features to the low-dimensional space. However, with the effect of rising dimensionality and dropout events, conventional similarity metrics have proven unreliable and inadequate in accurately capturing cell similarities. Moreover, PCA and t-SNE, not specifically designed for scRNA-seq data, may not be well-suited for complex and unknown scRNA-seq data distribution.

More recently, advancements in single-cell sequencing technology have spurred the development of complicated similarity measurement techniques and clustering algorithms for clustering cells. For example, Kiselev *et al.*[10] adopted the Euclidean, Pearson, and Spearman metrics to cells-related information to generate the consensus matrix for clustering. Method ENCORE[11] generally combined Entropy Subspace with consensus clustering, identifying low-entropy subspaces as informative and generating a similarity matrix for downstream analysis. However, these cell similarity-based approaches often only capture shallow relationships, making them struggle to effectively discover and exploit the high-order genetic variation features inherent in scRNA-seq data, thus limiting the ability to obtain cluster-friendly information with high confidence.

Deep learning approaches, particularly those leveraging autoencoders (AEs), have already demonstrated considerable efficacy in extracting meaningful representations for tasks like cell clustering based on gene expression profiles. For instance, scDeepCluster[12], developed by Tian et al., obtained a more robust feature representation by integrating noise reduction techniques into an autoencoder. sc-INDC[13] innovatively introduced virtual noise into a novel autoencoder model to mitigate the adverse effects of noise interference. However, AE-based methods often ignore the structural features of cells. To address this challenge, some GNN-based methods have emerged, which take into account the topological information that reveals the potential relationship between cells, including scGNN[14], scGDC[15], scGCL[16], etc. However, these approaches usually pass information between nodes without differentiating between noisy edges, and erroneous edges may mislead the clustering results.

Besides, some contrast learning-based methods also have attracted a great deal of attention. For example, Contrast-sc[17] first applied contrast learning to scRNA-seq for data analysis, extracting features by comparing similarities between cells. scDCCA[18] uses a combination of dual contrast modules and denoising auto-encoders to extract key features and to learn more cluster-friendly characteristics. However, contrast learning random negative sample sampling may introduce spurious negative samples that hinder high-quality cell clustering. Besides, the existing contrast learning methods often overlook the crucial knowledge of cell relationships in scRNA-seq data, which are integral to effective clustering.

Multi-view clustering is a common and foundational task across various domains such as machine learning[19], pattern recognition[20], and data mining[21]. A single view often fails to capture the full spectrum of data characteristics, whereas multiple views typically offer complementary information, facilitating a more comprehensive understanding of data objects and enabling deeper insights for cluster partitioning[22]. For example, MCGL[23] simultaneously utilized multiple types of cell-to-cell networks to provide a more comprehensive description of topological relationships. scMVFI[24] put forward a multi-view feature integration framework, which identified latent feature sets through intrinsic entropy models and constructs multiple network topologies with varying sampling rates. Some consensus clustering methods can be regarded as ensemble learning across views, but the lack of information-sharing mechanisms across views leads to clustering results that are not entirely satisfactory.

Despite significant progress in multi-view scRNA-seq data clustering, several challenges persist. Firstly, the high dimensionality and sparsity of scRNA-seq data pose a significant difficulty. Traditional dimensionality reduction methods often assume a lower-dimensional noise-free space, which may not accurately capture the complex, non-linear relationships within biological data. Deep learning approaches, such as the Zero Inflated Negative Binomial (ZINB) model [25], have emerged to address this challenge. For example, ZINB is a probability distribution in probability statistics and has been commonly used to deal with situations where there are a large number of zero values. However, the generalizability of these approaches to diverse scRNA-seq platforms remains under debate. Secondly, limitations arise when relying solely on a single feature space to capture cellular heterogeneity. While integrating information from complementary views, such as cell structure can enhance clustering accuracy, existing methods often struggle with efficient cross-view fusion mechanisms. Finally, dimensionality reduction techniques commonly employed in clustering, while aiming to preserve core information, often lead to inevitable information loss, potentially hindering the identification of cell types.

**Figure 1:**
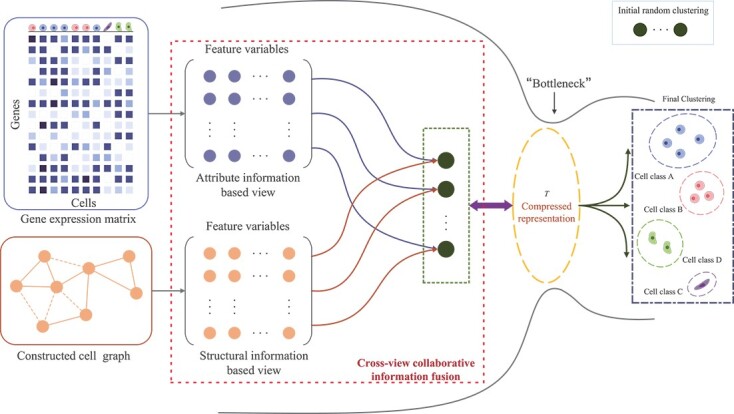
Architecture of scCFIB. Firstly, the attribute information based view and structural information based view are established respectively. Then the representations of cells are derived from these two views respectively. Finally, we adopt the IB strategy to perform the cross-view information fusion strategy, guiding the segregation of cell types and ultimately leading to the final clustering results. $T$ represents the final clustering result.

Motivated by the recognition of the significance of different views in the clustering process, we put forward a novel clustering approach with the cross-view collaborative information fusion strategy, which is presented in Fig. 1. This method aims to effectively utilize attribute and structural information to guide the cell clustering process and effectively address the high-dimensional and sparse challenge of clustering scRNA-seq data. The main contributions of our study are summarized as follows:

- scCFIB addresses the challenge of high-dimensional sparse data by leveraging the information bottleneck (IB) strategy. This allows the model to perform clustering directly on the original features, minimizing information loss.
- scCFIB constructs a multi-feature space and employs a powerful cross-view information fusion strategy to tackle the scRNA-seq clustering task.
- To handle the challenges of optimization in a multi-view setting, scCFIB introduces a novel sequential iterative optimization process, which improves the algorithm’s performance.
- The extensive evaluation of publicly available datasets demonstrates that the proposed scCFIB model outperforms other competitive methods in terms of clustering accuracy.

## Materials and methods

### The architecture of scCFIB

In this study, the scCFIB model takes the gene expression matrix $ R_{M \times N}$ as input, where the rows correspond to genes (features), and the columns describe cells (samples). Each element $ r_{ij} $ in the matrix represents the expression level of gene $ j $ in cell $i$.

The framework of the scCFIB algorithm has been illustrated in Fig. 1, which mainly includes three primary steps: construction of multi-view feature space, cross-view information collaboration strategy, and sequential optimization process.

Construction of multi-view feature space: the characteristic representations of cells are obtained from multiple views by the original gene expression matrix after preprocessing and the cell graph is generated based on the preprocessed matrix. At the same time, network enhancement (NE) is adopted to decrease the noise in the initial similarity matrix $ S_{n \times n} $ to obtain a more reliable graph structure $ A_{n \times n} $.

Cross-view information collaboration strategy: to synergistically integrate structural and attribute information and promote their mutual interactions, we improve the objective function of the IB method based on the constructed cellular graph to retain both attribute information and structural information of the scRNA-seq data.

Optimization process: a novel iteration strategy is devised to solve the optimization problem in the context of collaborative fusion, efficiently learning cell clustering results.

### Data description and preprocessing

In this study, we mainly collect 22 commonly used datasets from different biological tissues and sequencing platforms for experimental analysis and validation, with cell types derived from the original annotations. A detailed description of these datasets is shown in Table 1.

**Table 1:** Brief description of 22 experimental datasets used in the study

Datasets	Cells	Genes	Cell types	Sparsity	Platform	Reference
Biase	49	25 737	3	49.82%	Tang	[26]
Truetlein	80	20 124	5	90.20%	Smart-Seq	[27]
Yan	90	23 271	6	45.55%	Smart-Seq	[28]
Goolam	124	41 480	5	68.56%	Smart-Seq	[29]
Deng	268	22 431	6	60.46%	Smart-Seq	[30]
Buettner	182	8989	3	37.94%	Quartz-Seq	[31]
Pollen	301	23 730	11	67.14%	Smart-Seq	[32]
Darmanis	466	22 088	9	80.77%	Smart-Seq	[33]
Chung	515	57 915	5	90.00%	Microwell-Seq	[34]
Kolodziejczyk	704	10 685	3	27.87%	Smart-Seq	[35]
Sun.1	1756	1000	6	79.55%	Chromium	[36]
Baron-mouse	1886	14 878	13	88.97%	inDrop	[37]
Muraro	2122	19 046	9	73.02%	CEL-Seq2	[38]
Klein	2717	24 175	4	65.76%	inDrop	[39]
Romanov	2881	24 341	7	87.77%	Drop-Seq	[40]
10X PBMC	4271	16 653	8	92.24%	10X Genomics	[41]
Brown.1	4465	16 328	7	87.44%	Chromium	[42]
Sun.2	4853	1000	8	41.91%	Chromium	[36]
PBMC$^{1}$	5419	32 738	4	97.72%	Chromium	–
Sun.3	8218	1000	7	52.26%	Chromium	[36]
Well-paired-Seq PBMC	8027	14 566	13	97.43%	Well-paired-Seq	[43]
Mouse bladder	2746	20 670	16	94.87%	Microwell-Seq	[2]

$^{1}$ Dataset PBMC$^{1}$ from https://support.10xgenomics.com/single-cell-gene-expression/datasets/1.1.0/pbmc6k.

Specifically, the raw high-dimensional sparse scRNA-seq data $R_{M \times N}$ is preprocessed to the gene expression matrix $X_{m \times n}$. The preprocessing process mainly consists of three steps: gene filtering, normalization, and data log transformation respectively. Firstly, we apply quartiles to define thresholds for excluding low-quality genes, using 1.5 times the IQR above the upper quartile and below the lower quartile. Then, the gene expression matrix is normalized by dividing by the library size, multiplied by 10 000. Finally, the preprocessed matrix is generated by log-transformed with a pseudo count of 1. The normalization and log-transformation procedures can be formalized as follows: 


(1) \begin{align*} & x_{i j}=\log_{2}\left({\frac{x_{i j}}{\sum_{j=1}^{n}x_{i j}}}\times100000+1\right)\end{align*}

The detailed description of data collection and preprocessing is displayed in Supplementary Section 1.

### Graph construction based network enhancement strategy

The cell graph reflects the relationship between cell interactions. Considering both the expression profiles of cells and the complex topology between them when clustering can improve the clustering accuracy. For information sharing among similar cells, modeling cell-to-cell relationships from scRNA-seq promotes cell clustering, which requires the construction of a cell graph that integrates cellular neighbors over the entire population to explore cell relationships [44]. To construct the initial similarity matrix $S_{n \times n}$, the Pearson correlation coefficient between cells is calculated based on the matrix $ X_{m \times n}$ after preprocessing. The Pearson correlation coefficient between cell $ i $ and cell $ j $ is computed according to the following formula: 


(2) \begin{align*} & S_{i j}\,=\,{\frac{\sum_{k=1}^{m}(x_{k i}-{\bar{x}}_{i})\left(x_{k j}-{\bar{x}}_{j}\right)}{\sqrt{\sum_{k=1}^{m}\left(x_{k i}-{\bar{x}}_{i}\right)^{2}}\sqrt{\sum_{k=1}^{m}\left(x_{k j}-{\bar{x}}_{j}\right)^{2}}}}\end{align*}

where $k$ represents $k$th gene and $\bar{x_{i}} $ represents the mean of $i$th cell expression values.

Meanwhile, we utilize network enhancement(NE) to attenuate the noise effect and improve the quality of the constructed graphs to obtain more reliable clustering results. Taking the matrix $S_{n \times n}$ as input, scCFIB employs NE to obtain the enhanced matrix and performs the KNN algorithm to obtain the square matrix $ W$. The number of neighbors connected to each cell is determined by the $k$-nearest neighbor value. Finally, the matrix $ W $ is transformed into an undirected graph by 


(3) \begin{align*} & A_{i j}=\left\{ \begin{array}{@{}ll} {{1,}} & {{ P_{i j}>0}} \\{{0,}} & {{\mathrm{otherwise}}} \end{array}\right.\end{align*}

where $P = \frac{ 1} { 2 } ( W + W^ { T } )$. The matrix $ A $ represents the constructed cell graph $ A_{n \times n}$(ie. KNN graph), serving as the adjacency matrix for our generated undirected cell graph.

### IB-based collaborative fusion strategy

Cross-view clustering is a powerful technique for analyzing scRNA-seq data. Integrating multiple perspectives of the same biological system, can enhance information interoperability, optimize clustering structure, and improve model efficacy. The cross-view approach could offer a more comprehensive interpretation of scRNA-seq data, making it a valuable tool for understanding cellular heterogeneity.

IB was first proposed by Tishby based on rate-distortion theory, which treats data clustering as a data compression process[45]. The central idea of IB is to find a compressed representation $T$ for data object $ X $ and feature variables $Y$, maximizing information preservation while compressing $Y$ to faithfully represent the data features. The objective function of the IB method can be formulated as 


(4) \begin{align*} & {\mathcal{L}}_{\mathrm{max}}\left[p(t\mid x)\right]=I(T;Y)-\beta^{-1} I(T;X)\end{align*}

where the parameter $\beta $ is a Lagrange factor greater than or equal to 0, which is used to balance the compression of source information and the preservation of related information. $p(t \mid x)$ is the data compression pattern obtained by the IB method, i.e. the cluster label in the scenario of hard clustering. The mutual information between variables $X$ and $Y$ can be calculated as 


(5) \begin{align*} & I(X;Y)={\textstyle \sum_{x\in X}}{\textstyle \sum_{y\in Y}}p(x,y)\log{\frac{p(x,y)}{p(x)p(y)}}\end{align*}

A detailed description of the IB Information Theory used in this study is presented in Supplementary Section 3.

Based on the IB method and a cross-view framework, we construct two different data views, attribute information, and structural information, and redefine and characterize the single-cell clustering issue accordingly. Given one scRNA-seq dataset containing $ n $ cells $ X = \left \{x_{1},x_{2},\dots ,x_{n}\right \} $, each $ x_{i} $ corresponds to one cell. And it will be described using two views: gene attributes (view 1) includes $ m $ gene attribute features $ Y = \left \{y_{1},y_{2},\dots ,y_{m}\right \} $ taken from the gene expression matrix $ X_{m \times n}$ of the scRNA-seq data. The other is cell structure (view 2) includes structural features $ G = \left \{g_{1},g_{2},\dots ,g_{n}\right \} $ derived from the cell graph $ A_{n \times n} $. Raw features are used directly in the gene attributes view, with type labels assigned using the IB method, which effectively handles high-dimensional sparsity and avoids information loss from dimensionality reduction like PCA, resulting in more accurate clustering. Consequently, The goal of scCFIB is to derive the optimal clustering $ p(t|x) $ by integrating both views while preserving information from two related feature variables gene expression $ Y $ and cell relationship $ G $.

To employ comprehensively the intracellular gene expression information and intercellular relationship information for cell clustering, we propose a cross-view collaborative fusion strategy using the IB approach. Similar to traditional IB methods [46], scCFIB treats cell clustering as a ‘compression’ process, consisting of two parts: (1) Data Compression. The scRNA-seq data $X$ to be analyzed is transformed into the variable $T$ through compression, in the process minimizing the mutual information $I(T; X)$. (2) Information Preservation. Maximize the preservation of attribute and structural feature information to ensure that patterns inherent in scRNAseq data are represented as accurately as possible. Data compression and information preservation are formally described in the form of $I(T; X)$ and $I(T; Y)$, respectively.

Specifically, cells are initially randomly divided into $k$ clusters. The scCFIB algorithm iteratively extracts each cell from its current cluster and assigns it to the most suitable cluster, continuously repeating this process until cell clustering is achieved. During the allocation of each cell, the algorithm maximizes two different views of information: attribute information $I(T; Y)$ and structural information $I(T; G)$. This enables the fusion of information across views and guides cell separation collaboratively. Considering the information compression requirements of the IB method, the scRNA-seq data is compressed as much as possible under information preservation constraints, minimizing the mutual information between the final clustering result $T$ and the scRNA-seq data $X$. Therefore, the final cross-perspective collaborative fusion objective function which is the basis for the cell clusters is defined as follows: 


(6) \begin{align*} & {\mathcal{L}}_{m a x}=\alpha I(T;Y)+\left(1-\alpha\right)I(T;G)-\beta^{-1}I(T;X)\end{align*}

where $\beta \in \left ( 0, +\infty \right )$ is used to balance information preservation and data compression; The parameter $ \alpha \in \left [0,1 \right ] $ serves as a trade-off parameter that balances the contribution of attribute information $ Y $ and structural information $ G $ to cluster results.

### Optimation the strategy of scCFIB

The scCFIB algorithm optimizes the objective function using a sequential method within the framework of a collaborative fusion strategy. scCFIB starts with a random initialization partition $ T = \left \{t_{1},t_{2},\dots ,t_{k}\right \} $ with $ k $ clusters. Afterward, two processes of iterative ‘drawn’ and ‘merged’ processes are used to find the best cluster for each cell $ x_{i} \in X $. In the ‘drawn’ step, each cell $ x_{i} $ is first extracted from its current cluster $ t_{old} $ and treated as a separate cell cluster. To maintain a partition with specific $ k $ clusters, in the ‘merged’ step, we need to merge the separate cell $ x_{i} $ into the optimal cluster $t_{new}$ to form a new cluster $\hat{t}$. the optimization process continues until the cell groups are stable or a set number of iterations is reached. Guided by the greedy algorithm, the goal of the scCFIB algorithm is to maximize the objective function by merging each cell $ x_{i} \in X $ into the optimal cluster to form a new partition $ T^{new} $. The optimization process for cross-views collaborative fusion can be formalized as the following formula: 


(7) \begin{align*} & \left\{ \begin{array}{@{}ll} {p({\hat{t}})=p(x)+p(t)} \\{p(y|{\hat{t}})={\frac{p(x)}{p(\hat{t})}}\cdot p(y|x)+{\frac{p(t)}{p(\hat{t})}}\cdot p(y|t)} \end{array} \right.\end{align*}

The matter of this process is how to maximize the objective function by selecting the right candidate cell clusters during the fusion process. When the cell $ x $ is drawn or merged from the existing clusters to create new clusters, it alters the value of the objective function. We set the value of the objective function after the ‘drawn’ process as $ \mathcal{L}_{bef} $, and the value of the objective function of the new partition $ T^{new} $ after the ‘merged’ process as $ \mathcal{L}_{atf} $. The fusion loss can then be formalized as the change in the value between $ \mathcal{L}_{atf} $ and $ \mathcal{L}_{bef} $, and this is the basis for selecting the new cluster: 


(8) \begin{align*} \notag{} & \kern-2pc d_{\mathcal{L}}(\{x\},t) =\mathcal{L}^{bef}-\mathcal{L}^{aft} \\ \notag ={} & \left[ \alpha I(T^{bef};Y) + (1- \alpha)I(T^{bef};G)-\beta^{-1}I(T^{bef};X))\right ] \\ \notag & -\left [ \alpha I(T^{atf};Y) + (1-\alpha)I(T^{atf};G)-\beta^{-1}I(T^{atf};X)\right ] \\ \notag ={} & \alpha \left [ I(T^{bef};Y) - I(T^{atf};Y) \right ] + (1- \alpha) \\ \notag & \left [ I(T^{bef};G) -I(T^{atf};G)\right ]-\beta^{-1}\left [ I(T^{bef};X) - I(T^{atf};X) \right ] \\ ={} & \alpha \Delta I_{1}+(1-\alpha)\Delta I_{2}-\beta^{-1}\Delta I_{3}\end{align*}

The alteration in the objective function value is referred to as the ‘merged cost’. According to Equation(7), the following results can be obtained: 


(9) \begin{align*} \notag{} &\kern-2pc \Delta I_{1}=I\left(T^{bef};Y\right)-I\left(T^{atf};Y\right) \\ \notag ={} & p(x)\sum_{y\in{\mathcal{Y}}}p\left(y|x\right)\log{\frac{p(y|x)}{p(y)}}+p(t)\sum_{y\in{\mathcal{Y}}}p\left(y|t\right)\log{\frac{p(y|t)}{p(y)}} \\ \notag & -p(\hat{t})\sum_{y\in{\mathcal{Y}}}p\left(y|\hat{t}\right)\log{\frac{p(y|\hat{t})}{p(y)}} \\ \notag ={} & p(x)\sum_{y}p(y|x)\log\frac{p(y|x)}{p(y|\hat{t})}+p(t)\sum_{y}p(y|t)\log\frac{p(y|t)}{p(y|\hat{t})} \\ \notag ={} & p(x)D_{KL}\left[ p(y|x)|| p(y|\hat{t})\right ]+ p(t)D_{KL}\left[ p(y|t)|| p(y|\hat{t})\right ] \\ ={} & p(\hat{t})\cdot{JS}_{\mathrm{\Pi}}\left[ p(y|x), p(y|t)\right ]\end{align*}

where $ {JS}_{\mathrm{\Pi }}= \pi _{1} D_{KL}\left [ p(y|x)|| p(y|\hat{t})\right ] +\pi _{2} D_{KL}\left [ p(y|t)|| p(y|\hat{t})\right ] $ and $ \Pi =\{\pi _{1},\pi _{2}\}=\{\frac{p(x)}{p(\hat{t})},\frac{p(t)}{p(\hat{t})}\} $

Similarly, we can get 


\begin{align*} \notag \Delta I_{2} & = p(\hat{t})\cdot{JS}_{\mathrm{\Pi}}\left[ p(g|x),p(g|t)\right ] \\ \notag \Delta I_{3} & = p(\hat{t})\cdot{JS}_{\mathrm{\Pi}}\left[ p(x),p(x|t)\right]\end{align*}

where $ JS $ denotes *Jensen-Shannon* divergence and $ D_{KL} $ denotes *Kullback-Leibler* divergence.

Since $ JS $ is non-negative, the target value before and after each ‘drawn-merged’ process satisfies $ \mathcal{L}_{atf} \ge \mathcal{L}_{bef} $, which can be obtained as follows $ d_{\mathcal{L}}(\{x\},t) \ge 0 $. This means that in the process of fusing to a new cell cluster, some loss of information is bound to be faced. Therefore, in the fusion phase, the optimal clustering results are chosen for each cell $x_{i} \in X $ formulated by $ t_{new}= \arg min_{t \in T}{d_{\mathcal{L}}(\{x\},t)} $ so that the gene expression information and structural information of cells are maximized and preserved.

A detailed description of the Optimization process of scCFIB is shown in Supplementary Section 4.

Algorithm 1 summarizes the optimization process of scCFIB.


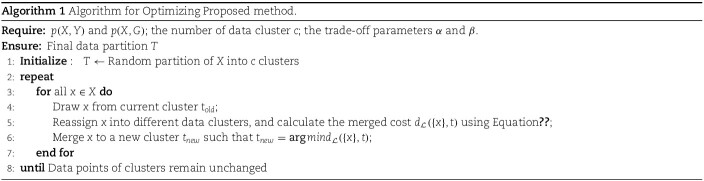


### Evaluation indicators

Four widely-used evaluation metrics in single-cell experiments, Adjusted Rand Index(ARI)[47] and Normalized Mutual Information(NMI)[48], Adjusted Mutual Information(AMI)[49], Fowlkes–Mallows Index(FMI)[50], are utilized to evaluate the performance of scCFIB alongside other comparison methods. These metrics are defined as follows: 


(10) \begin{align*} & \mathrm{ARI}\,= \frac{RI-E\left[RI\right]}{max\left[ RI-E\left[ RI\right] \right]} \end{align*}

(11) \begin{align*} & \mathrm{NMI}\,= \,{\frac{I(P,Q)}{\big[ H(P)+ H(Q)\big]/2}} \end{align*}

(12) \begin{align*} & \mathrm{AMI}\,= \frac{MI-E\left[MI\right]}{\big[H(P)+ H(Q)\big]/2-E\left[ MI\right]} \end{align*}

(13) \begin{align*} & \mathrm{FMI}\,= \frac{TP}{\sqrt{(TP+FP)\times(TP+FN)}} \end{align*}

The ARI is a modified version of the Rand Index for random assignment, providing a more fair assessment of clustering results. In Equation(10), the Rand Index measures segmentation similarity based on correct and incorrect object assignments. NMI evaluates the quality of clustering algorithms by quantifying the amount of information shared between the clustering results and the ground truth labels. In Equation(11), $\mathrm{P} = \{ P_{ 1}, P_{ 2 }, \cdots , P_{ k } \}$ and $\mathrm{Q} = \{ Q_{ 1 }, Q_{ 2 }, \cdots , Q_{ k } \}$, respectively, represent the cell clusters predicted by the algorithm and real cell types. $ H(\cdot )$ is the entropy function and $ I(P,Q) $ represents the mutual information of $ P $ and $ Q $. AMI is an adjustment of the Mutual Information score to account for chance, and the AMI values range from −1 to 1. FMI is the Fowlkes–Mallows score, defined as the geometric mean of the precision and recall of the pairwise. In Equation(13), TP represents the true cases shared between clusters in real labels and clustering results. FP denotes false positives, and FN signifies false negatives. The above four metrics range from −1 to 1. The larger the value, the better the clustering results. A detailed description of the evaluation metrics is presented in Supplementary Section 2.

## Results

### Comparison with other baselines

To validate the effectiveness of the algorithms, we compare scCFIB with eight scRNA-seq clustering algorithms on 22 publicly available datasets. The eight selected methods for comparison can be categorized into three categories: traditional methods, AE-based deep learning methods, and graph-based deep learning methods. A brief description of these eight baselines is shown in Supplementary Section 5.

**Figure 2:**
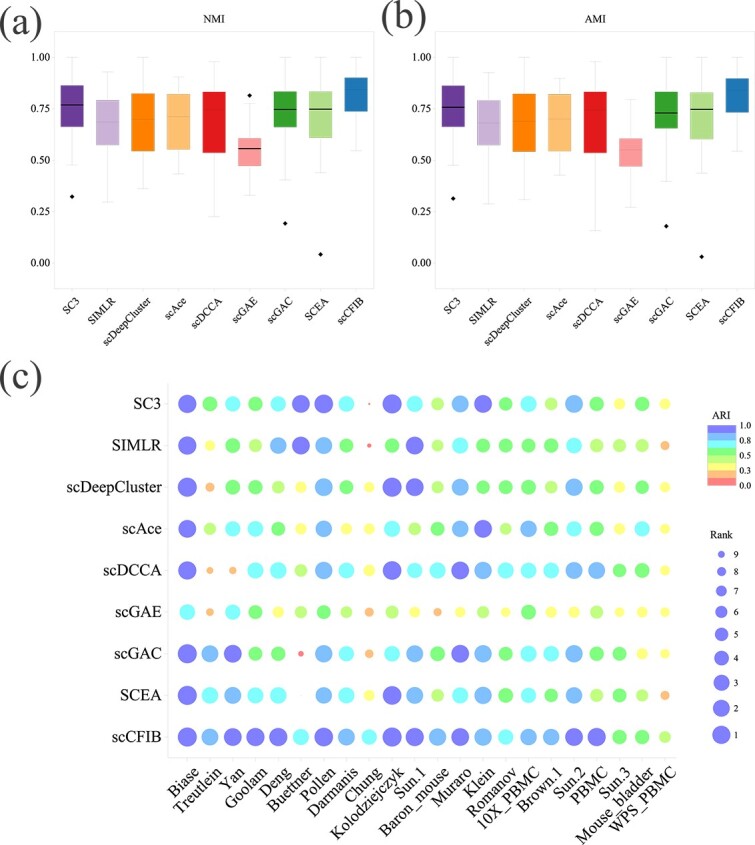
Comparison results of different approaches across 22 scRNA-seq datasets. (a) Box plots of NMI scores on 22 datasets. (b) Box plots of AMI scores. (c) The Bubble plots of ARI scores for nine approaches on various datasets, where WPS PBMC refers to Well-paired-Seq PBMC.

As expected, scCFIB demonstrates consistent and outstanding performance across all evaluation metrics, as illustrated in Fig. 2 and Table 2. The box plot reflects the overall distribution of data, while the bubble plot can visually observe the size and ranking of the data. Results indicate that scCFIB is not only at a leading level overall among the average clustering results of 22 datasets, but also has more stable clustering results. Moreover, the clustering results of scCFIB have a competitive advantage compared to the other eight algorithms. Given the biological complexity inherent in the datasets, it’s acknowledged that no single method can universally perform optimally across all datasets and scenarios. Nonetheless, scCFIB consistently ranks within the top three among all compared methods, showcasing remarkable performance in clustering tasks across nearly all datasets (Shown in Table 2). Among them, it achieves the highest FMI metrics on 17 datasets. The aforementioned findings collectively demonstrate that our method surpasses the performance of the eight compared methods in clustering. In addition, we find that the scCFIB algorithm exhibits good clustering performance on highly sparse datasets such as Treutlein, Chung, and Baron mouse, demonstrating that the IB-based approach has a strong ability to handle high-dimensional sparsity.

**Table 2:** FMI scores of scCFIB and baseline methods on 22 datasets, the bold values indicate the top 3 results

Datasets	SC3	SIMLR	scDeepCluster	scAce	scDCCA	scGAE	scGAC	SCEA	scCFIB
Biase	0.9669	0.9669	**1.0000**	0.9277	0.9642	0.7890	**1.0000**	**1.0000**	**1.0000**
Treutlein	0.7329	0.4956	0.4303	0.5604	0.3753	0.3888	**0.8657**	**0.8287**	**0.8705**
Yan	0.7279	0.6884	0.6995	0.7378	0.3098	0.7303	**0.9147**	**0.8522**	**0.9172**
Goolam	0.7063	0.6321	0.7173	0.7872	**0.8453**	0.6704	0.6950	**0.8121**	**0.9544**
Deng	0.7560	**0.8533**	0.5998	0.6655	**0.8307**	0.5283	0.6844	0.8274	**0.9398**
Buettner	**0.9348**	**0.9254**	0.6236	0.5403	0.6749	0.6196	0.5711	0.5640	**0.8127**
Pollen	**0.9627**	0.8064	**0.8844**	0.8008	0.8655	0.5975	0.8505	0.7879	**0.9650**
Darmanis	0.7487	0.6176	0.6264	0.4818	**0.7493**	0.4969	0.7473	**0.7843**	**0.8545**
Chung	0.3606	0.3875	0.5237	0.5529	**0.5854**	0.4980	0.5155	**0.5637**	**0.7835**
Kolodziejczyk	**1.0000**	0.7348	**1.0000**	0.8158	0.9913	0.6814	0.8006	0.9983	**1.0000**
Sun.1	0.7635	**0.9047**	**0.9085**	0.5965	0.7302	0.4691	0.8459	0.8376	**0.9437**
Baron-mouse	0.5802	0.5367	0.5636	0.6723	**0.7842**	0.3643	**0.7072**	0.6396	**0.8832**
Muraro	0.8565	0.7986	0.8519	0.8304	**0.916**	0.4372	**0.9065**	0.8163	**0.9139**
Klein	**0.9318**	0.6818	0.6740	**0.9109**	0.9083	0.5732	0.8836	0.8965	**0.9092**
Romanov	0.6327	0.6556	0.6521	0.5142	**0.7747**	0.4266	0.6495	**0.6994**	**0.7444**
10X PBMC	**0.7696**	0.6393	0.6724	**0.8133**	0.7571	0.6773	0.7657	0.7333	**0.8220**
Brown.1	0.5800	0.7021	0.5812	0.6950	**0.7753**	0.5068	**0.8019**	0.6951	**0.8509**
Sun.2	**0.8941**	0.7560	0.8594	0.7155	0.8545	0.4724	**0.8609**	0.8196	**0.9055**
PBMC	0.7393	0.7200	0.7347	0.7396	**0.8961**	0.6775	**0.7645**	0.6969	**0.9617**
Sun.3	0.5223	0.6514	0.5172	0.5274	**0.6911**	0.4611	**0.6932**	0.6748	**0.6983**
Mouse bladder	0.6160	0.5307	0.5726	**0.7126**	**0.6738**	0.4063	0.4597	0.5710	**0.6320**
Well-paired-Seq PBMC	**0.4130**	0.3279	0.3729	**0.4591**	0.3788	0.3317	0.3847	0.3849	**0.4594**
Average	0.7362	0.6824	0.6848	0.6844	0.7424	0.5365	**0.7440**	**0.7493**	**0.8555**

**Figure 3:**
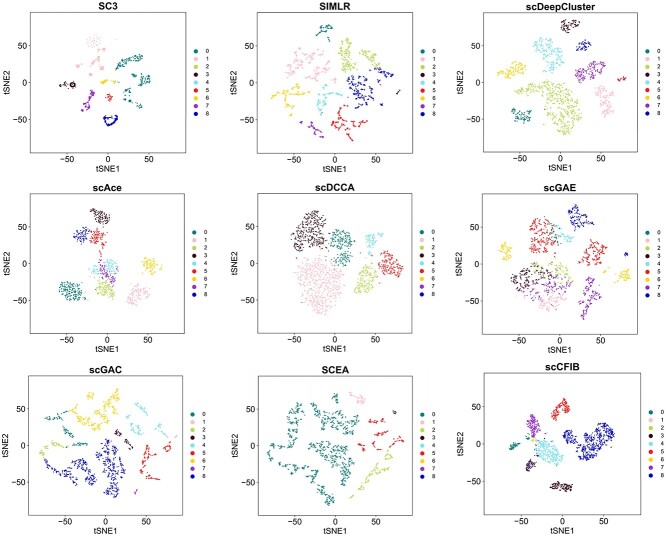
Clustering performance of scCFIB and the baselines with tSNE on the Muraro dataset.

Finally, to demonstrate the intuitive clustering effect of scDCCA, we conduct the cell visualization experiment on the Muraro dataset with t-SNE. The results are presented in Fig. 3. The boundaries of various cell subtypes of scCFIB are clearer than those from the other methods and the samples in the clustering results are more concentrated, implying its effectiveness and reliability.

Moreover, the comparison results of scCFIB and other baselines under the V-Meansure metric are presented in Supplementary Table S1. The ARI score of scCFIB and baseline methods on all the datasets are displayed in Supplementary Table S6. The visualization results of scCFIB and other baselines under NMI and AMI metrics are shown in Supplementary Figure S1 and Figure S2 respectively.

### Analysis of the effect of NE on scCFIB

To investigate the effect of the cellular relationship graph constructed after NE enhancement on the clustering results, we perform an experiment on all datasets again. We want to compare the difference in final clustering performance under a single view and across views using NE technology and not using NE technology. ARI is used to assess the accuracy of clustering. One follows the standard process of scCFIB and the other removes the NE technique at the stage of constructing the cell graph.

**Figure 4:**
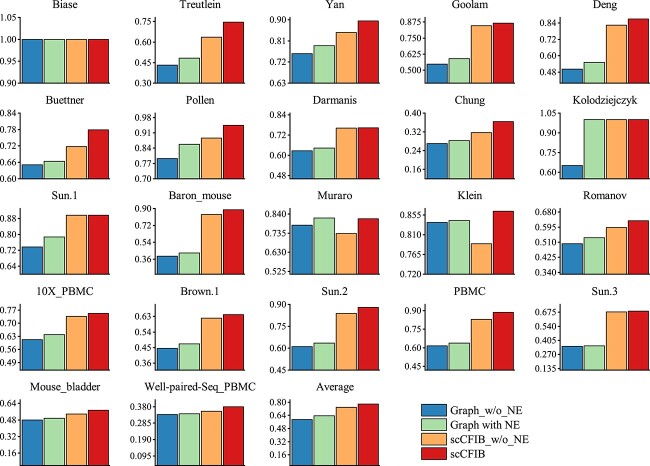
Analyze the effect of NE in single-view and cross-view, respectively. The graph records the ARI scores of Graph_w/o_NE, Graph_NE, scCFIB_w/o_NE, and scCFIB under four different experimental settings of NE.

To ensure fairness and reliability, all the comparison models utilize the same preprocessing. The experiment was set up in four situations: Graph_w/o_NE, Graph_NE, scCFIB_w/o_NE, and scCFIB. The first two situations, respectively, represent the conditions of using and not using NE when scCFIB removes the attribute perspective and only uses the cell structure perspective, that is, the NE experimental comparison under a single structural information view. The last two situations, respectively, represent the conditions of using and not using NE when both attribute and structure information are used, that is, the NE experimental comparison under cross-view. The result is shown in Fig. 4 that the graph constructed using NE technology improves the accuracy of clustering and can significantly improve downstream analysis. This phenomenon suggests that the ability of aggregating neighbors to preserve structural information between cells is crucial for clustering.

In single-cell clustering, graph construction is often based on the measure of cell-cell similarity. Nonetheless, owing to the characteristics of high-dimensional sparse single-cell sequencing data, the quality of the constructed graph frequently falls short of expectations. To verify the reliable improvement and effectiveness of NE on the construction graph, we will use the matrix after NE denoising and the graph of the primordial cell similarity matrix. To understand the effect of NE noise reduction on the reliable improvement of construction graphs and its effectiveness, We use the proportion of correct edges in the constructed cell graph as an indicator to qualitatively analyze and verify the role of NE. The higher the proportion of correct edges, the stronger the reliability of the constructed graph. To this end, we compare the primordial cell similarity matrix with the matrix after denoising using NE to see if they have the expected block diagonal structure. Ideally, the similarity of different types of cells is 0, and the similarity between the same types of cells is 1. Therefore, the clearer the structure of the block diagonal submatrix means the better clustering results will be obtained.

**Figure 5:**
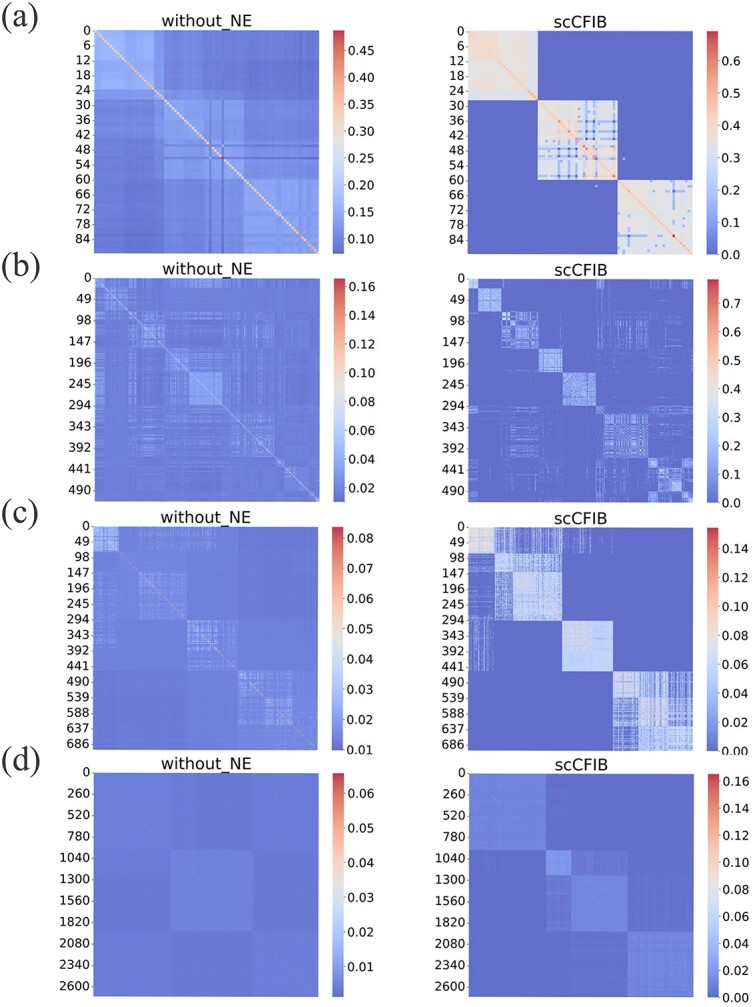
Visualization of cell similarity matrix with or without NE, where without_NE and scCFIB represent the construction of cell graphs without NE and using NE technology, respectively. (a) Yan dataset, (b) Chung dataset, (c) Kolodziejczyk dataset, (d) Klein dataset.

Table 3 provides the results of the correct edge ratio of graphs constructed with and without NE for 22 datasets. Except for the Yan dataset, the proportion of correct edges in graphs enhanced by NE on other datasets is higher than that using only Pearson coefficients for composition, indicating that NE is beneficial in constructing cell graphs. As shown in Fig. 5, we visualize the four datasets of Yan, Chung, Kolodziejczyk, and Klein using the similarity matrix of NE and not using NE denoising. Compared with the result without NE denoising, the result shows a clear block diagonal structure after NE denoising, while without NE denoising is fuzzy and irregular. This phenomenon indicates that the use of NE can indeed enhance the similarity between the same cells, weaken the noise edge, and make the boundaries between different cells clearer. In summary, the cell graph constructed by NE denoising is more reliable and robust, which is beneficial to clustering cells.

**Table 3:** Proportion of correct edges in constructed cell graphs without or with NE strategy

Datasets	without NE	with NE	Datasets	without NE	with NE
Biase	**100%**	**100%**	Baron-mouse	83.9%	**85.1%**
Treutlein	62.0%	**71.3%**	Muraro	78.0%	**79.0%**
Yan	**91.5%**	91.3%	Klein	83.8%	**83.9%**
Goolam	**95.6%**	**95.6%**	Romanov	81.6%	**83.9%**
Deng	**90.2%**	90.1%	10X PBMC	66.1%	**71.2%**
Buettner	68.3%	**69.0%**	Brown.1	73.0%	**75.9%**
Pollen	84.7%	**85.4%**	Sun.2	95.8%	**95.9%**
Darmanis	81.1%	**82.8%**	PBMC	92.6%	**94.2%**
Chung	87.1%	**88.5%**	Sun.3	79.5%	**80.1%**
Kolodziejczyk	98.8%	**99.7%**	Mouse bladder	64.1%	**65.7%**
Sun.1	94.1%	**94.5%**	Well-paired-Seq PBMC	34.4%	**37.8%**

### Biological analysis

To prove that the results of scCFIB clustering have biological significance, we conducted two experiments: gene differential expression analysis and marker gene identification, which is simiar to scDCCA[18]. Based on the results predicted by scCFIB and the true cell types, we searched for differentially expressed genes between cell clusters to help identify cell types. Specifically, we first used the Wilcoxon Rank Sum test[51] to identify the top 50 differentially expressed genes for each cluster and cell type in the three datasets of Darmanis, Muraro, and Sun.1, and then calculated the overlap rate of differentially expressed genes between each cell cluster and each true type. Finally, we assigned the cell type corresponding to the maximum overlap rate of each cluster to the cluster to achieve cell annotation.

For example, in the Darmanis dataset, the cell clusters (0–8) predicted by scCFIB correspond to the real cell types oligodendrocytes, hybrid, fetal_quiescent, OPC, endothelial, astrocytes, fetal_replicating, and microglia. Experiments conducted on multiple datasets (shown in Supplementary Tables 2–4) demonstrated that the clustering results of scCFIB are biologically meaningful and can effectively separate different cell types.

**Figure 6:**
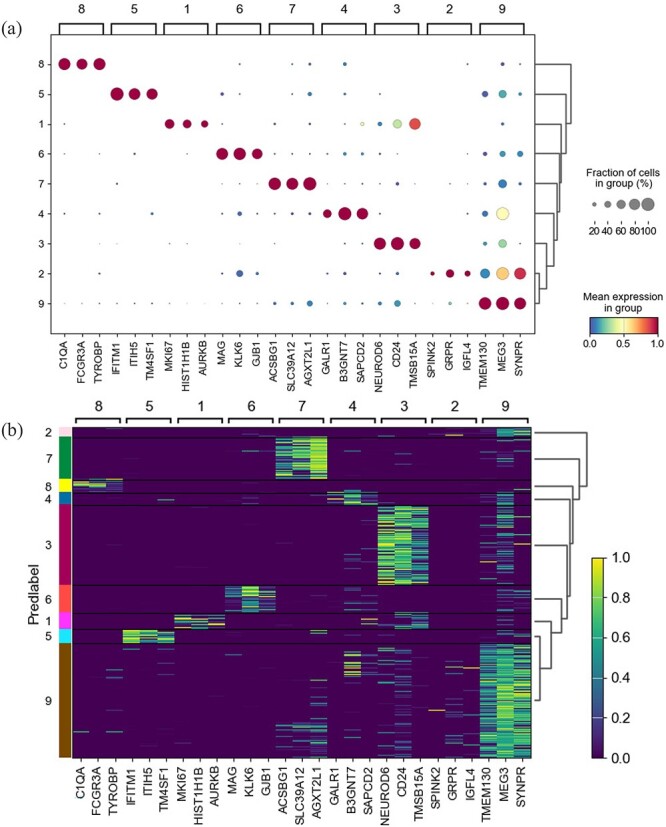
Expression dot plot and heatmap of the top three marker genes identified for each cell type by scCFIB. (A) Expression dot plots (The color of the dot indicates the mean expression value of the gene within each cluster, and the size of the dot indicates the fraction of cells in each group expressing the gene); (B) Expression heat maps.

In addition, for the Darmanis dataset, we selected the top 3 differentially expressed genes in each cluster for visualization to help observe the expression levels of genes in different cell clusters and identify marker genes for each cluster predicted by scCFIB. The results of Fig. 6 show that these genes are more significantly expressed in specific cell clusters than in other cell clusters. To verify whether these marker genes are real and reliable, we compared them with the marker genes recorded in the database CellMarker[52]. As a result, most of the genes marked by scCFIB prediction results are consistent with those known marker genes recorded in the CellMarker database. For example, the genes in cluster 6 predicted by scCFIB (MAG, KLK6, GJB1) are marker genes of oligodendrocyte cells, which shows that the cell clusters predicted by scCFIB are true and accurate. In summary, the labels generated by scCFIB assist in the identification of marker genes and enable precise annotation of cell types.

### Ablation study

In this study, our model inputs the characteristics of cells from two different views: gene expression matrix and cell relationship graph. Because of the high-dimensional characteristics of scRNA-seq data, some dimensionality reduction methods and feature selection methods to select highly variable genes are usually used to reduce the dimension of scRNA-seq data. Although it can improve the efficiency of clustering to a certain extent, it inevitably leads to the loss of information, resulting in poor clustering results. In addition, the choice of dimension reduction is also a problem to be considered. Therefore, we retain all the information in the gene expression matrix to solve the problem of information loss in the process of feature selection. To fully leverage the topological information of the data, scCFIB constructs a KNN graph using nearest neighbor information between cells, thus guiding the algorithm to achieve superior clustering results. Furthermore, to demonstrate the superior performance of the IB algorithm in clustering, we will compare its effectiveness with Kmeans and Leiden, two conventional algorithms commonly utilized in single-cell clustering, specifically under the single-view data.

**Figure 7:**
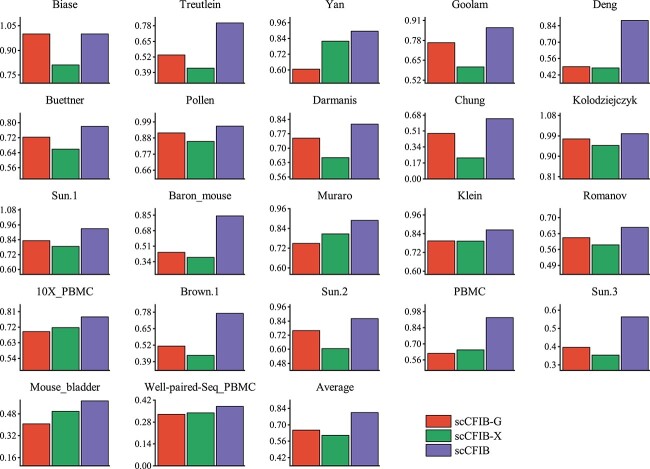
Ablation experiment analysis. Compare the clustering results of scCFIB under a single view with those under a cross-view fusion strategy. The experimental results of scCFIB are superior to using a single view, indicating that an effective cross-view fusion strategy can improve clustering performance.

**Figure 8:**
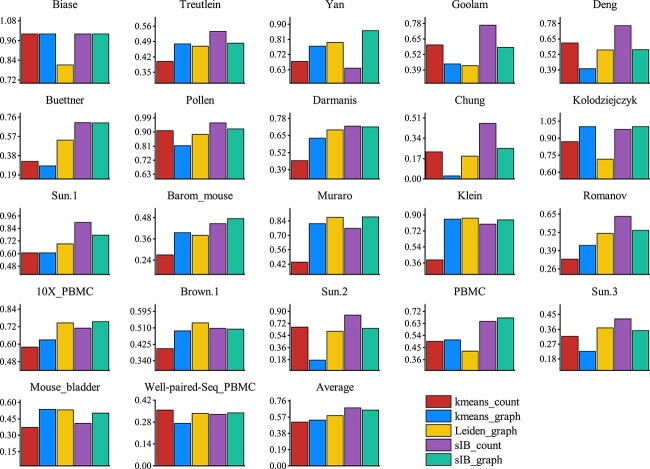
The performance of Kmeans, Leiden, and sIB, respectively. Leiden is the graph clustering algorithm and only applies to cell graphs, while Kmeans and sIB could be used on both gene matrix and cell graphs. ARI is adopted as the evaluation indicator.

To illustrate the cooperation between the two views and independently show the contribution of the gene expression matrix and cell graph, we devise three distinct experiments. The integration of the gene expression matrix and the cell graph, utilizing either the raw count matrix alone or solely the cell graph. Specifically, we set the parameter $ \alpha $ to 0 (ie, scCFIB-G) when we only use the expression matrix and $ \alpha $ to 1 (ie, scCFIB-X) when we use only the cell graph. ARI is employed to assess the clustering outcomes of all experiments. As depicted in Fig. 7, it is evident that the performance of data utilizing only a single view across various datasets is both unstable and subpar. The combination of the two views, scCFIB, can obtain the best clustering results. This observation highlights the importance of simultaneously integrating attribute information and structural information and the effectiveness of the cross-view fusion strategy. Combining information from both views allows us to leverage the strengths of each view, resulting in superior clustering performance. Additionally, as observed in Fig. 8, when utilizing data from a single view, Kmeans and Leiden’s methods are dominant in some datasets, but the IB-based method achieves the best average clustering effect, whether it is a single gene expression matrix or a cell graph. The results show that IB can effectively handle high-dimensional sparse data and has natural advantages in clustering such data. Therefore, it is a suitable choice to use the IB method as a basis for studying cell clustering problems.

### Parameter sensitivity analysis

In this section, we discuss the parameter sensitivity, mainly involving two parameters $ \alpha $ and $ \beta $. Two important parameters $ \alpha $ and $ \beta $ are used to balance information preservation and data compression in the IB clustering process and the contribution of two different views, respectively. Here, we explore the impact of two hyperparameters on the performance of scCFIB individually. We set $ \alpha $ to $ \{0.1, 0.3, 0.5, 0.7, 0.9\}$, $ \beta $ is $\{10, 100, 500, 700, 900\}$. Grid search is adopted to explore the clustering performance under different parameter settings of $ \alpha $ and $ \beta $.

**Figure 9:**
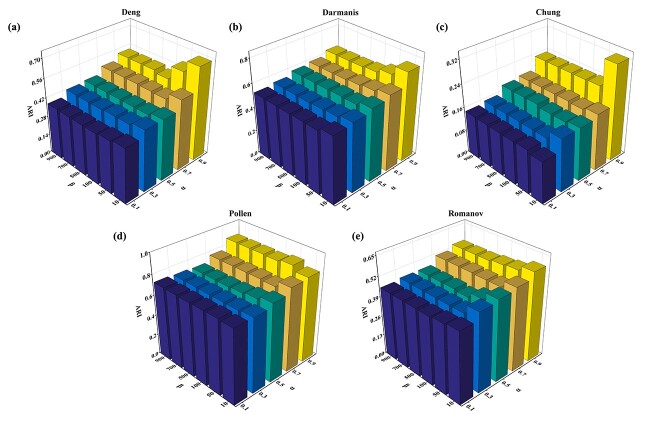
Parameter sensitivity analysis results. The ARI values of scCFIB with different settings on five experimental datasets.

Taking the Deng dataset and the other four datasets as an example, as shown in Fig. 9, it is obvious that under the same $ \alpha $ setting, with the increase of $ \beta $, the clustering index ARI hardly fluctuates. When $ \beta = [10, 50]$, the best clustering effect is often obtained. The clustering performance is greatly affected by $\alpha $, and the clustering index ARI increases with the increase of $\alpha $, and the best clustering effect is obtained in $\alpha =0.9$. The results show that when a suitable $ \alpha $ is given, the attribute information and structure information can be well integrated into the scCFIB and promote the clustering results. Therefore, the proposed scCFIB method is robust enough to the variation of hyperparameters.

### Scalability and efficiency analysis

Scalability is the capacity of an algorithm to perform efficiently with increasing amounts of data. As single-cell sequencing technology advances, data volumes will grow, making algorithm scalability essential for effective data handling and analysis. Here we evaluate scCFIB’s scalability and efficiency using two large datasets, Chen [53] and CD19[54], each with over 10 000 cells. Chen has 14437 cells and 23 284 genes with 47 cell types, while CD19 has 10 085 cells and 32 738 genes with 10 cell types. The detailed information is displayed in the Supplementary Table S5.

The clustering results using four metrics across datasets for scCFIB and the baselines are presented at Table 4. The results show that scCFIB exhibits competitive clustering results even on large datasets, indicating that scCFIB has the good scalability.

**Table 4:** Performance of scCFIB and baselines on CD19 and Chen datasets

**Algorithm**	**CD19**				**Chen**			
	**ARI**	**NAMI**	**AMI**	**FMI**	**ARI**	**NMI**	**AMI**	**FMI**
SC3	0.2220	0.3240	0.3220	0.4791	0.3733	0.6486	0.6298	0.4662
SIMLR	0.1192	0.1872	0.1857	0.4444	0.3388	0.5805	0.5708	0.4308
scDeepCluster	0.1699	0.2764	0.2753	0.3713	0.5045	0.6795	0.6728	0.5740
scAce	0.1729	0.3197	0.3193	0.5604	0.6500	0.7003	0.6987	0.7128
scDCCA	0.2234	0.2984	0.2973	0.4832	0.6914	0.7312	0.7285	0.7427
scGAE	0.0717	0.1784	0.1771	0.2424	0.2306	0.5905	0.5810	0.3323
scGAC^*^	0.1015	0.2185	0.2175	0.5353	—	—	—	—
SCEA^*^	0.0350	0.1113	0.1106	0.5348	—	—	—	—
scCFIB	0.1920	0.3270	0.3259	0.5424	0.6931	0.7130	0.7069	0.7405

**—** indicates the missing values. Since the Chen dataset is too large, scGAC and SCEA methods are unable to run and obtain the results.

Besides, we also display the running time of baselines on the different experimental datasets. The results shows that scCFIB outperforms scGAC and SCEA in runtime but still faces efficiency issues. We categorize datasets as small datasets(<2000 cells) and large datasets(>2000 cells), analyzing runtime for each group. Table 5 indicates scCFIB ranks third in efficiency on small datasets, performing well, but struggles with large datasets, taking more time. Furthermore, the memory usage for scCFIB is medium.

**Table 5:** The average running time of the nine representative approaches on all the experimental datasets and their corresponding memory consumption

**Algorithm**	**Running Time(s)**		**Memory(MB)**
	**Cells<2,000**	**Cells>2,000**	
SC3	561.00	4140.55	1088.26
SIMLR	1185.71	507.55	3328.85
scDeepCluster	252.35	2396.27	101.92
scAce	416.64	3068.26	3526.64
scDCCA	43.46	395.35	2077.38
scGAE	69.14	2478.14	3467.17
scGAC^*^	1624.11	57760.44	85.21
SCEA^*^	1113.07	60074.98	87.15
scCFIB	143.52	10868.42	677.81

^*^scGAC and SCEA didn’t contain the running time on the Chen dataset.

In order to show the clustering results more intuitive and validate the effectiveness of our scCFIB. We adopt t-SNE to visualize the cluster results and true labels on three datasets. The results are presented in Supplementary Section 6.

Finally, we analyze the time complexity of scCFIB. A given expression matrix $R_{M \times N}$, contains $ N $ cells and $ M $ genes. $ X $ represents all of these $ N $ cells. Suppose the cells are divided into $ k $ clusters. The main time of the algorithm is spent in the optimization process. In step 4 of the optimization algorithm 1, the distance between the data object and the $ JS $ distribution of the centroid of each cluster. The time complexity of this step is $ O(k) $. Therefore, the time complexity of steps 3 to 7 is $ O(k|X|) $. The overall time complexity of the algorithm is $ O(Lk|X|)$, where $ L $ denotes the number of iteration cycles required for the scCFIB algorithm to converge to a locally optimal solution. Therefore, the time complexity of the scCFIB algorithm is linearly related to the size of the data.

A limitation of scCFIB is its runtime and memory consumption rate, both of which are in the middle of the evaluation dataset, and run less efficiently than algorithms such as SC3, SIMLR, etc., but compared to scDCCA, scAce has a good memory consumption. Therefore, in the future, we consider optimizing the running efficiency and resource consumption of the algorithm to better apply it to larger datasets.

## Conclusion

We propose scCFIB, a cross-view information fusion method based on IB. It initially uses IB to handle high-dimensional sparse features and directly clusters original features to minimize information loss from dimensionality reduction. Then, it constructs a cell graph to incorporate cell topological structure information, considering both expression patterns and cell interactions. Our algorithm realizes cross-view collaborative information fusion and compression of attribute and structural information based on IB, retaining both types of information to achieve better clustering. Experimental results demonstrate that effective information fusion promotes high-quality clustering results.

In the future, we will involve enhancing the algorithm through improved cell auxiliary graph construction and incorporating relevant data. High-quality cell graphs greatly influence clustering accuracy, necessitating the pursuit of more robust construction methods. Additionally, optimizing multi-view architecture by integrating diverse data types aims to innovate and expand research horizons.

**Key Points**

- scCFIB leverages the information bottleneck (IB) method to address the challenge of high-dimensional sparse data. This approach allows the model to perform clustering directly on the original features, thereby minimizing information loss.
- scCFIB constructs a multi-feature space and employs a novel cross-view information fusion strategy to facilitate information integration between views.
- To address the optimization challenges inherent to multi-view learning, scCFIB incorporates a novel sequential iterative optimization process, which improves its clustering performance.
- Experimental results demonstrate that our proposed model outperforms all baseline methods in the clustering results.

## Supplementary Material

Supp_final_version_bbae511

## Data Availability

The experimental datasets and code of scCFIB used in this manuscript are available through https://github.com/weixiaojiao/scCFIB.
