# Supplementary material for: Clustering scRNA-seq data with the cross-view collaborative information fusion strategy
Source: Brief Bioinform. 2024 Oct 14;25(6):bbae511. doi: 10.1093/bib/bbae511 (PMC11473192; doi:10.1093/bib/bbae511)
Supplement: Supp_final_version_bbae511 [file supp_final_version_bbae511.pdf]

Supplementary Figures

Supplementary Figure S1

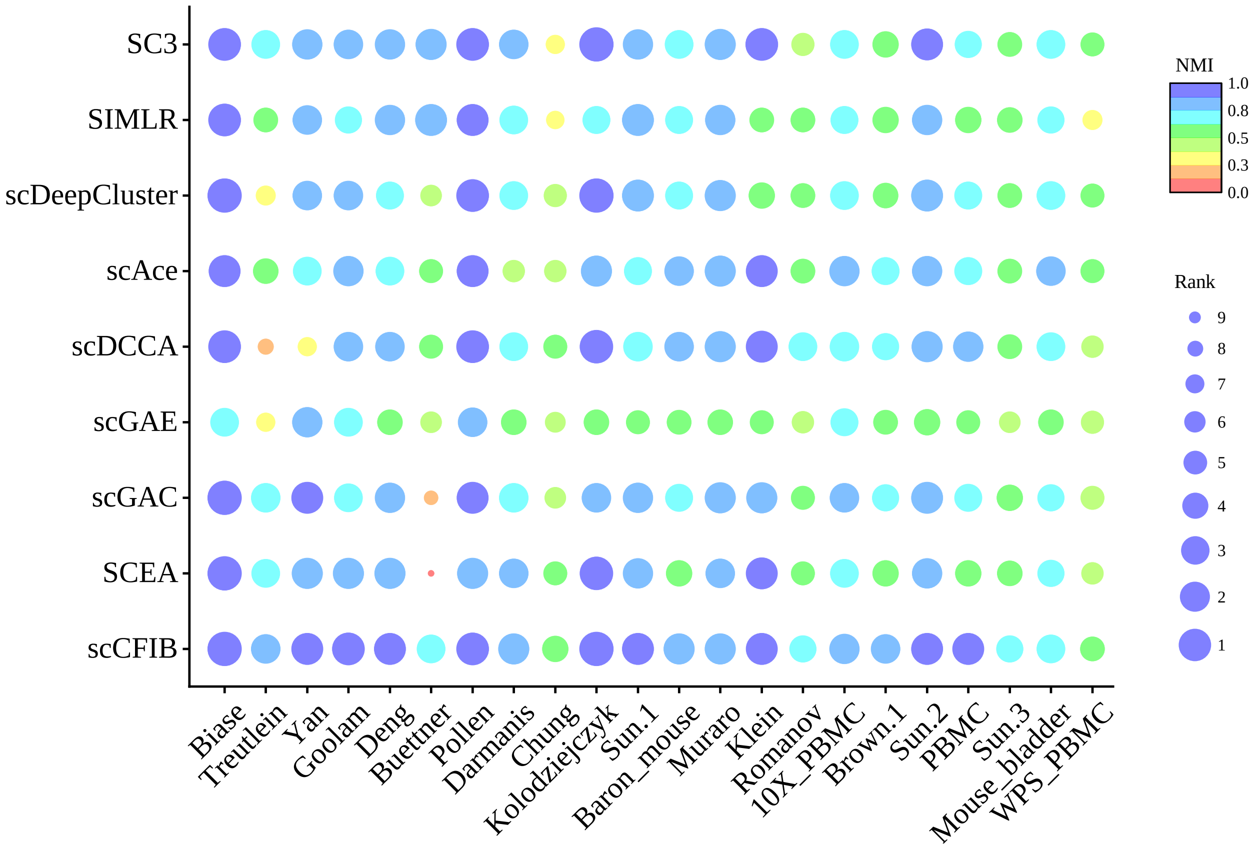

. Figure S1. NMI scores of scCFIB and 8 baseline methods on 22 evaluated datasets. A bubble represents the performance of methods, where the size indicates the rank and the color indicates the NMI score. And the WPS_PBMC refers to Well-paired-Seq_PBMC.

Supplementary Figure S2

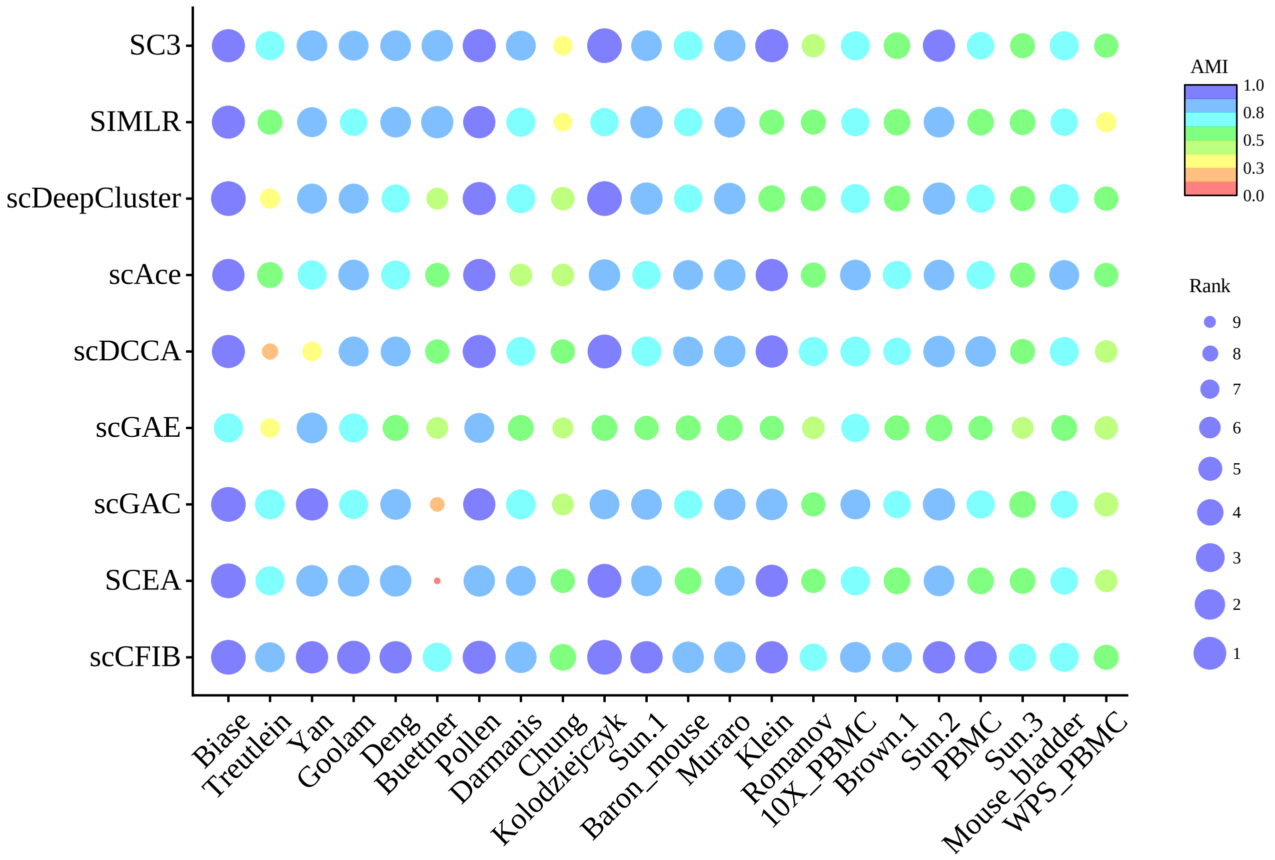

. Figure S2. AMI scores of scCFIB and 8 baseline methods on 22 evaluated datasets. A bubble represents the performance of methods, where size indicates the rank and the color indicates the AMI score. And the WPS_PBMC refers to Well-paired-Seq_PBMC.

Supplementary Tables

Supplementary Table S1

Table S1. The results of scCFIB and baseline methods on 22 datasets with V-Measure metric. The bold value indicates the top 3 results.

Datasets	SC3	SIMLR	scDeepCluster	scAce	scDCCA	scGAE	scGAC	SCEA	scCFIB
Biase	0.9293	0.9293	**1.0000**	0.9048	0.9334	0.7051	**1.0000**	**1.0000**	**1.0000**
Treutlein	0.7083	0.5264	0.3627	0.5631	0.2264	0.3281	**0.7484**	**0.7271**	**0.7665**
Yan	0.7951	0.7695	0.7525	0.7144	0.3336	0.8141	**0.9012**	**0.8527**	**0.9056**
Goolam	0.7583	0.6550	0.7634	**0.7984**	0.7715	0.7195	0.7385	**0.8274**	**0.9163**
Deng	0.7840	**0.7982**	0.6818	0.7100	0.7646	0.5774	0.7890	**0.8338**	**0.8917**
Buettner	**0.8917**	**0.8744**	0.4039	0.5066	0.5113	0.4203	0.1929	0.042	**0.7195**
Pollen	**0.9534**	0.8851	**0.9256**	0.8869	0.9223	0.7750	0.8923	0.8648	**0.9369**
Darmanis	**0.7786**	0.7188	0.7041	0.4417	0.7236	0.5719	0.7453	**0.7821**	**0.8476**
Chung	0.3225	0.2969	0.4690	0.4335	**0.5029**	0.3777	0.4038	**0.5006**	**0.6149**
Kolodziejczyk	**1.0000**	0.6993	**1.0000**	0.8271	0.9795	0.5646	0.7684	0.9950	**1.0000**
Sun.1	0.7858	**0.8662**	**0.8663**	0.6767	0.7468	0.5220	0.8079	0.7997	**0.8976**
Baron-mouse	0.7265	0.6960	0.6919	**0.7595**	**0.7810**	0.5484	0.6947	0.6097	**0.8390**
Muraro	**0.8534**	0.7920	0.8239	0.8341	**0.8395**	0.5808	0.8335	0.7590	**0.8395**
Klein	**0.9255**	0.5563	0.6145	0.8870	**0.8969**	0.5189	0.8592	0.8770	**0.8975**
Romanov	0.4763	**0.5506**	0.5442	0.5467	**0.7123**	0.4435	0.5212	0.5178	**0.6401**
10X_PBMC	0.7390	0.6736	0.7290	**0.7882**	0.7453	0.6667	**0.7535**	0.7375	**0.7844**
Brown.1	0.5941	0.5955	0.5915	**0.6779**	0.6552	0.5467	**0.6607**	0.6089	**0.7506**
Sun.2	**0.8860**	0.7857	**0.8723**	0.8217	0.8323	0.6062	0.8696	0.8192	**0.9008**
PBMC	0.6626	0.6154	0.6710	0.6734	**0.8131**	0.5217	**0.6743**	0.6157	**0.8755**
Sun.3	0.5243	0.5739	0.5356	0.5523	0.5363	0.4028	**0.5989**	**0.5770**	**0.6287**
Mouse_bladder	0.7268	0.6617	**0.7369**	**0.7761**	0.7348	0.5868	0.6620	0.6442	**0.7375**
Well-paired-Seq_PBMC	**0.5142**	0.3675	**0.5111**	0.5075	0.4567	0.4718	0.4955	0.4392	**0.5461**
Average	**0.7412**	0.6767	0.6932	0.6949	0.7009	0.5577	**0.7096**	0.7014	**0.8153**

Supplementary Table S2

Table S2. The assignments of cell types to predicted clusters for the Darmanis dataset.

Cell Type	Cell cluster	Overlap ratio	Unique gene in predicted cluster	Unique gene in cell type
microglia	8	0.82	CD84, CSF1R, FGD2, etc.	BCL2A1, CXorf21, HLA-DRB4, etc.
endothelial	5	1.00		
oligodendrocytes	1	0.84	CENPK, DMRTA2, HIST1H1D, etc.	DACH1, IQGAP2, LIPG, etc.
astrocytes	6	0.90	ADAMTS18, HSPA2, NINJ2, etc.	AIF1L, APOD, OLIG1, etc.
fetal_replicating	7	0.98	MT1E	RHOJ
OPC	4	0.68	AFAP1L2, FAM5C, FLJ31485, etc.	C3AR1, CMTM7, DAB2, etc.
fetal_quiescent	3	0.96	MAP2, SEMA3A	NFIB, VASH2
neurons	9	0.84	CNTNAP2, GAD1, GPRASP1, etc.	B3GNT1, CHGA, NCDN, etc.

Supplementary Table S3

Table S3. The assignments of cell types to predicted clusters for Muraro dataset.

Cell Type	Cell cluster	Overlap ratio	Unique gene in predicted cluster	Unique gene in cell type
mesenchymal	1	0.68	A2M, EMP1, FMNL3, etc.	BASP1, COL5A3, CRLF1, etc.
beta	5	0.90	DOCK10, PIR, PPP2R2C, etc.	ATRNL1, CSDE1, EIF4A2, etc.
delta	4	0.66	ABCC9, AQP3, CARD11, etc.	AMIGO2, ANK1, ASB4, etc.
alpha	9	0.94	COX8A, CPE, TUSC3, etc.	GPR64, POPDC3, SPOCK3, etc.
pp	8	0.88	APCS, HSPB8, KCNK5, etc.	ABCC3, DAB2, EPB41L4A, etc.
duct	6	0.96	BCL2L15, GJB1	PLA2G1B, PTF1A

Supplementary Table S4

Table S4. The assignments of cell types to predicted clusters for Sun.1 dataset.

Cell Type	Cell cluster	Overlap ratio	Unique gene in predicted cluster	Unique gene in cell type
Neutrophils	2	1.00		
Club Cells	4	0.98	Crip2	Tmem252
Small Airway Epithelial Cells	5	0.98	Rpl17	Rplp0
Endothelial Cells	6	0.94	Fos, Lgals1, Sub1	Rpl10, Rps19, Rps7
Lymphocytes	1	0.90	Ddx27, Fmo2, Lcn2, etc.	Cystm1, Dbi, Perp, etc.
Macrophages	3	0.98	Cox6c	Adk

Supplementary Table S5

Table S5. The brief description for dataset Chen and CD19.

Datasets	Sequencing platform	Cells	Genes	Groups	Sparsity
CD19	10X Genomics	10085	32738	10	98.38%
Chen	Drop-seq	14437	23284	47	93.36%

Supplementary Table S6

Table S6. ARI score of scCFIB and baseline methods on 22 datasets, the bold value indicates the top 3 results.

Datasets	SC3	SIMLR	scDeepCluster	scAce	scDCCA	scGAE	scGAC	SCEA	scCFIB
Biase	0.9483	0.9483	**1.0000**	0.8900	0.9445	0.6786	**1.0000**	**1.0000**	**1.0000**
Treutlein	0.6099	0.3055	0.2276	0.4033	0.1340	0.1769	**0.7937**	**0.7436**	**0.8044**
Yan	0.6584	0.6117	0.6164	0.6297	0.1508	0.6625	**0.8923**	**0.8112**	**0.8955**
Goolam	0.5728	0.4709	0.5875	**0.6825**	0.7400	0.5244	0.5538	**0.7176**	**0.9296**
Deng	0.6530	**0.7741**	0.4534	0.5359	**0.7489**	0.3563	0.5586	0.7455	**0.9122**
Buettner	**0.9025**	**0.8882**	0.3450	0.3700	0.4592	0.4098	0.0884	0.0028	**0.7181**
Pollen	**0.9581**	0.7826	**0.8698**	0.7606	0.8418	0.5450	0.8289	0.7593	**0.9602**
Darmanis	**0.6962**	0.5428	0.5534	0.3450	0.6861	0.3878	0.6591	**0.7155**	**0.8164**
Chung	0.0118	0.0522	0.2922	0.3269	**0.3714**	0.2399	0.2160	**0.3280**	**0.6413**
Kolodziejczyk	**1.0000**	0.5644	**1.0000**	0.7206	0.9866	0.4893	0.6959	0.9973	**1.0000**
Sun.1	0.7068	**0.8812**	**0.8858**	0.4387	0.6631	0.3253	0.8065	0.7963	**0.9289**
Baron-mouse	0.4344	0.3862	0.4144	**0.5532**	**0.7074**	0.1980	0.6027	0.4547	**0.8403**
Muraro	**0.8134**	0.7416	0.8078	0.7807	**0.8909**	0.2998	0.8785	0.7101	**0.8882**
Klein	**0.9007**	0.5272	0.5045	**0.8761**	0.8627	0.4050	0.8356	0.8491	**0.8644**
Romanov	0.5103	0.5493	0.5436	0.3775	**0.7041**	0.2560	0.5326	**0.5931**	**0.6578**
10X_PBMC	**0.7155**	0.5496	0.6073	**0.7567**	0.6757	0.6054	0.7102	0.6651	**0.7793**
Brown.1	0.4294	0.5665	0.4336	0.5770	**0.6655**	0.3437	**0.7020**	0.5136	**0.7701**
Sun.2	**0.8687**	0.6995	0.8260	0.6386	0.8113	0.3493	**0.8270**	0.7769	**0.8789**
PBMC	0.5652	0.4916	0.5581	0.5666	**0.8121**	0.4614	**0.5826**	0.4700	**0.9280**
Sun.3	0.3504	0.4828	0.3461	0.3585	0.5082	0.2606	**0.5549**	**0.5328**	**0.5634**
Mouse_bladder	0.5602	0.4623	0.5100	**0.6413**	**0.6072**	0.3262	0.3668	0.4096	**0.5737**
Well-paired-Seq_PBMC	**0.3307**	0.2292	**0.2976**	0.2686	0.2730	0.2608	0.2660	0.2240	**0.3794**
Average	**0.6453**	0.5685	0.5764	0.5681	**0.6475**	0.3892	0.6342	0.6280	**0.8059**

Supplementary Section

Supplementary Section 1: Data description and preprocessing

In this study, the experimental datasets are available through the GEO database or websites. The datasets and accession numbers obtained from the GEO website are Biase (GSE57249), Chung(GSE75688), Sun.1/2/3(GSE128066), Brown.1(GSE137710), Well-paired-Seq_PBMC (GSE192708), Chen(GSE87544). In addition, datasets available via the website are The Mouse_bladder dataset originates from the Mouse Cell Atlas project (https://figshare.com/s/865e694ad06d5857db4b), the Darmanis and Buttener dataset can be downloaded from (https://github.com/BatzoglouLabSU/SIMLR/tree/SIMLR/data), the 10X_PBMC was obtained at https://github.com/ttgump/scDeepCluster/tree/master/scRNA-seq%20data. The other datasets are downloaded from https://hemberglab.github.io/scRNA.seq.datasets.

We collected a total of 22 publicly available scRNA-seq datasets that are used for experimental analysis and validation. Details of these data sets are described below:

Biase[1] contains 49 mouse embryonic cells belonging to three cell types: 2-cell mouse embryos, 4-cell mouse embryos, and zygotes.

Treutlein [2] dataset is derived from mouse lung epithelial cells and contains 80 cells, 23,271 features, and 4 cell types.

Yan[3] dataset includes 124 individual cells from human pre-implantation embryos and human embryonic stem cells by applying the single-cell RNA-seq technique.

Goolam[4] is Single-cell RNA-seq data of blastomeres from 2- to 32-cell stage mouse embryos, according to Developmental stage can be divided into cleavage 4-cell, cleavage 2-cell, cleavage 8-cell, cleavage 16-cell, cleavage 32-cell.

Deng[5] dataset contains 268 mouse cells at different stages of development, classified into 6 different cell types: blast, 2cell, 4cell, 8cell, 16cell, and zygote.

Buettner[6] dataset is the single-cell RNA-seq data from a population of staged mouse embryonic stem cells (mESCs) using the Fluidigm C1 protocol. It includes the transcriptional profile of 182 mESCs that have been staged for the cell-cycle phase (G1, S, and G2M).

Pollen[7] dataset was constructed by 301 single cells from 11 populations in developing cerebral cortex using microfluidics and analyzing single-cell transcriptomes across downsampled sequencing depths.

Darmanis[8] dataset is single-cell RNA sequencing data on 466 cells, which captures the cellular complexity of the adult and fetal human brain at a whole transcriptome level. Individual cells can be able to classify into all of the major neuronal, glial, and vascular cell types in the brain.

Chung[9] dataset is the single-cell mRNA expression profiles which acquired epithelial tumor and tumor-infiltrating immune cells from eleven patients (BC01-BC11) including two lymph node metastases (BC03LN, BC07LN).

Kolodziejczyk[10] dataset is the single cell RNA-sequencing data of Mouse embryonic stem cells (mESCs) cultured in three different conditions: serum, 2i, and the alternative ground state a2i.

Sun[11] dataset includes sequencing results from peripheral blood mononuclear cells and skin cells from humans, as well as lung cells from mice. In this study, Sun.1 represents Mouse lung cells, Sun.2 represents Human skin cells, and Sun.3 represents Human peripheral blood mononuclear cells.

Baron_mouse[12] dataset contains 1,886 cells, which are obtained from two strains of mice, enriched for endocrine cells (islets), and identified 13 different cell populations.

Muraro[13] dataset is the transcriptome of thousands of single pancreatic cells from donors, allowing in silico purification of all main pancreatic cell types.

Klein[14] dataset consists of sequencing results from Mouse embryonic stem cells of four cell types.

Romanov[15] dataset is mouse hypothalamus cells and is sorted into major types (1001 oligodendrocytes, 267 astrocytes, 356 ependymal cells, 48 microglial cells, 240 endothelial cells, 71 vascular and smooth muscle lineage cells, and neurons).

10X_PBMC[16] dataset includes 4271 fresh peripheral blood mononuclear cells(PBMCs), with eight different cell types, which is then filtered to 4197.

Brown[17] dataset is the sequencing result performed on CD45 positive cells in melanoma tumors from two patients. where Brown.1 is the Human dendritic cells of the Brown dataset.

PBMC (`https://support.10xgenomics.com//single-cell-gene-expression//datasets//1.1.0//pbmc6k`) dataset includes 5356 Human peripheral blood mononuclear cells, eight different cell types, and then it is filtered to 5356.

Mouse_bladder[18] dataset obtained from the Mouse Cell Atlas project(`https://figshare.com/s/865e694ad06d5857db4b`), and is a total of 2746 single-cell transcriptomes of mouse bladder cells, which is then filtered to 2669.

Well-paired-Seq_PBMC[19] dataset obtained by Well-paired-seq sequencing, containing about 20000 cells, filtered out cells expressing less than 200 genes, resulting in 8027 cells.

Raw scRNA-seq data are high-dimensional sparse data filled with a lot of noise. Quality control and preprocessing are required before clustering can be performed[20]. The raw gene expression matrix $R_{M\times N}$ is preprocessed to $X_{m\times n}$ after quality control and normalization. The preprocessing consists of three steps, which are gene filtering, normalization, and data log-transformation respectively.

First, we adopt quartiles to set upper and lower threshold values which are set to the upper quartile adding 1.5 times IQR and the lower quartile minus 1.5 times IQR respectively. If the library size and gene expression number are outside the threshold (below the lower limit, or above the upper limit), it means that it may be a low-quality cell or a housekeeping gene that is stably expressed in all cells, which is seldom helpful for clustering cells and may even cause some negative effects. Therefore, these cells and genes need to be removed to ensure that the gene expression values are distributed within a reasonable range. Then, the gene expression matrix is normalized by dividing by the library size, multiplied by 10000. Finally, the preprocessed matrix is generated

by log-transformed with a pseudo count of 1. The normalization and log-transformation procedures can be formalized as follows:

$$x_{ij} = \log_2\left(\frac{x_{ij}}{\sum_{j=1}^{n} x_{ij}} \times 100000 + 1\right) \tag{1}$$

Supplementary Section 2: Evaluation metrics

In addition to the four metrics Adjusted Rand Index(ARI)[21] and Normalized Mutual Information(NMI)[22], Adjusted Mutual Information(AMI)[23] and Fowlkes-Mallows Index(FMI)[24].

The adjusted Rand Index (ARI) is a modified version of the Rand Index (RI) that corrects for the effects of random assignment, thereby providing a more fair assessment of clustering results. Its formula is as follows:

$$\text{ARI} = \frac{RI - E\left[RI\right]}{max\left[RI - E\left[RI\right]\right]} \tag{2}$$

where RI, the Rand Index, is an object-based assignment indicator that measures the degree of similarity of data segmentation by the ratio of the number of correctly assigned and incorrectly assigned objects. The ARI value ranges from -1 to 1, where its larger value implies better clustering results.

Based on the concept of information theory, NMI mainly uses mutual information to measure the similarity between two sets (real labels and clustering results) to compare the similarity between clustering results and known true labels. NMI is calculated as follows:

$$\text{NMI} = \frac{I(P, Q)}{\left[H(P) + H(Q)\right]/2} \tag{3}$$

where P $= \{P_1, P_2, \cdots, P_k\}$ and Q $= \{Q_1, Q_2, \cdots, Q_k\}$ respectively represent the cell clusters predicted by the algorithm and real cell types. $H(\cdot)$ is the entropy function and $I(P, Q)$ represents the mutual information of P and Q. NMI is in the range of [0,1], where larger values indicate a closer alignment between the clustering results and the real labels, reflecting a better match between them.

Adjusted Mutual Information (AMI) is an adjustment of the Mutual Information (MI) score to account for chance. It accounts for the fact that the MI is generally higher for two clusterings with a larger number of clusters, regardless of whether there is more information shared. For the clusters and labels, the AMI is given as:

$$\text{AMI} = \frac{MI - E\left[MI\right]}{\left[H(P) + H(Q)\right]/2 - E\left[MI\right]} \tag{4}$$

This metric is independent of the absolute values of the labels: a permutation of the class or cluster label values won't change the score value in any way.

$$\text{FMI} = \frac{TP}{\sqrt{(TP + FP) \times (TP + FN)}} \tag{5}$$

In addition, we additionally supplemented the clustering results with V-Meansure[**?**]. Homogeneity and completeness are mutual information scores based on conditional entropy to measure the similarity between vectors, and the V-Measure is a reconciled average of homogeneity and completeness, calculated as follows:

$$\mathcal{V} = \frac{(1 + \beta) \times HI \times CI}{\beta \times HI + CI} \tag{6}$$

Where HI is homogeneity and CI is completeness, both taking values in the range of [0,1]. V-Measure integrates homogeneity and completeness and can evaluate the quality of clustering results more comprehensively. The higher its value, the better the quality of the clustering results. When the V-Measure is 1, it indicates that the clustering results are perfect, i.e., completely homogeneous and completely complete.

Supplementary Section 3: IB Information Theory

Information bottleneck (IB)[25] is first proposed by Tishby based on rate-distortion theory, which treats data clustering as a data compression process. The central idea of IB is to seek a compressed representation T (i.e., the bottleneck variable T) for a given data object X and feature variables Y describing the data object, maximizing the preservation of the information contained in the feature variables while compressing them as much as possible to more accurately reflect the intrinsic patterns of the data. The objective function of the IB method can be formulated as:

$$\mathcal{R}(D) = \min_{\{p(t|x):I(T;Y)\leq D\}} I(T;X) \tag{7}$$

Where $p(t|x)$ is the compression variable T, i.e., the optimal coding scheme, or clustering result, given the information preservation constraints are met. D is all possible coding schemes. $I(T;X)$ and $I(T;Y)$ are the amount of information between the two variables used to guide the 'compression' process, calculated as follows:

$$I(X;Y) = \sum_{x\in X}\sum_{y\in Y} p(x,y)\log\frac{p(x,y)}{p(x)p(y)} \tag{8}$$

Reference [1], the IB objective function can be rewritten via the Lagrange multiplier method as:

$$\mathcal{L}_{\max}\left[p(t\mid x)\right] = I(T;Y) - \beta^{-1}I(T;X) \tag{9}$$

Supplementary Section 4: Optimization process of scCFIB

To optimize the objective function, a sequential method is devised to address optimization problems within the framework of a collaborative fusion strategy. scCFIB starts with a random initialization partition $T = \{t_1, t_2, \ldots, t_k\}$ of X, where k represents the number of cell clusters. Then it iterates through two steps. In each step, each cell $x_i \in X$ is first drawn from the cluster t_{old} to which it currently belongs and treated as a separate cell cluster. At this time, there are $k+1$ clusters. To maintain a partition with specific k clusters, in the next step, we need to merge the drawn cell x_i into the optimal cluster t_{new}. The target of the scCFIB algorithm is to maximize the objective function, so a suitable cell cluster t_{new} must be selected for each cell for joining. With the help of the idea of the greedy algorithm, the best fusion cluster from appropriate cardinality is selected to form a new partition T^{new} based on the objective function. Each cell x_i is incorporated into the optimal cluster t_{new} to form a new cluster $\hat{t}$, and the optimization process continues until the cell groups are stable or a set number of iterations is reached. The fusion process can be formalized as the following formula:

$$\begin{cases} p(\hat{t}) = p(x) + p(t) \\ p(y|\hat{t}) = \frac{p(x)}{p(\hat{t})} \cdot p(y|x) + \frac{p(t)}{p(\hat{t})} \cdot p(y|t) \end{cases} \tag{10}$$

The matter of this process is how to maximize the objective function by selecting the right candidate cell clusters during the fusion process. When the cell x is drawn or merged from the existing clusters to create new clusters, it alters the value of the objective function. We set the value of the objective function after the 'drawn' process as $\mathcal{L}_{bef}$, and the value of the objective function of the new partition T^{new} after the fusion process as $\mathcal{L}_{atf}$. The fusion loss can then be formalized as the change in the value between $\mathcal{L}_{atf}$ and $\mathcal{L}_{bef}$, and this is the basis for selecting the new cluster:

$$\begin{aligned}
&d_{\mathcal{L}}(\{x\}, t) = \mathcal{L}^{bef} - \mathcal{L}^{aft} \\
&= \left[\alpha I(T^{bef}; Y) + (1-\alpha) I(T^{bef}; G) - \beta^{-1} I(T^{bef}; X))\right] \\
&\quad - \left[\alpha I(T^{atf}; Y) + (1-\alpha) I(T^{atf}; G) - \beta^{-1} I(T^{atf}; X)\right] \\
&= \alpha \left[I(T^{bef}; Y) - I(T^{atf}; Y)\right] + (1-\alpha) \\
&\quad \left[I(T^{bef}; G) - I(T^{atf}; G)\right] - \beta^{-1} \left[I(T^{bef}; X) - I(T^{atf}; X)\right] \\
&= \alpha \Delta I_1 + (1-\alpha)\Delta I_2 - \beta^{-1} \Delta I_3
\end{aligned} \tag{11}$$

The alteration in the objective function value is referred to as the 'merged cost'.

$$\begin{aligned}
&\Delta I_1 = I\left(T^{bef}; Y\right) - I\left(T^{atf}; Y\right) \\
&= p(x) \sum_{y \in \mathcal{Y}} p\left(y|x\right) \log \frac{p(y|x)}{p(y)} + p(t) \sum_{y \in \mathcal{Y}} p\left(y|t\right) \log \frac{p(y|t)}{p(y)} \\
&\quad - p(\hat{t}) \sum_{y \in \mathcal{Y}} p\left(y|\hat{t}\right) \log \frac{p(y|\hat{t})}{p(y)}
\end{aligned} \tag{12}$$

According to Eq.(10), the following results can be obtained:

$$\begin{aligned}
&\Delta I_1 \\
&= p(x) \sum_y p(y|x) \log \frac{p(y|x)}{p(y)} + p(t) \sum_y p(y|t) \log \frac{p(y|t)}{p(y)} \\
&\quad - \sum_y p(x) p(y|x) \log \frac{p(y|\hat{t})}{p(y)} - \sum_y p(t) p(y|t) \log \frac{p(y|\hat{t})}{p(y)} \\
&= p(x) \sum_y p(y|x) \log \frac{p(y|x)}{p(y|\hat{t})} + p(t) \sum_y p(y|t) \log \frac{p(y|t)}{p(y|\hat{t})} \\
&= p(x) D_{KL}\left[p(y|x) || p(y|\hat{t})\right] + p(t) D_{KL}\left[p(y|t) || p(y|\hat{t})\right] \\
&= p(\hat{t}) \cdot JS_{\Pi}\left[p(y|x), p(y|t)\right]
\end{aligned} \tag{13}$$

where $JS_{\Pi} = \pi_1 D_{KL}\left[p(y|x)||p(y|\hat{t})\right] + \pi_2 D_{KL}\left[p(y|t)||p(y|\hat{t})\right]$ and $\Pi = \{\pi_1, \pi_2\} = \{\frac{p(x)}{p(\hat{t})}, \frac{p(t)}{p(\hat{t})}\}$
Similarly, we can get:

$$\Delta I_2 = p(\hat{t}) \cdot JS_{\Pi}\left[p(g|x), p(g|t)\right]$$

$$\Delta I_3 = p(\hat{t}) \cdot JS_{\Pi}\left[p(x), p(x|t)\right]$$

Where JS denotes *Jensen-Shannon* divergence and D_{KL} denotes *Kullback-Leibler* divergence.

Supplementary Section 5: Baseline methods introduction

We compare this with eight algorithms, of which a brief description of each is given below:

SC3[26]: uses the consensus clustering strategy to fuse the clustering results obtained from multiple methods and generates a consensus matrix to obtain stable and robust results.

SIMLR[27]: uses multi-kernel learning and graph diffusion techniques to learn appropriate distance measures for the similarity between cells and employs spectral clustering for grouping cells.

scDeepCluster[28]: combines the advantages of DCA and DEC methods. Denoising techniques are introduced in the ZINB model-based autoencoder to obtain more accurate feature representations.

scAce[29]: uses a variational encoder to simultaneously learn cell embedding and cluster allocation. Through enhanced initialization, it achieves improved clustering performance, enhancing accuracy without requiring prior estimation of the number of clusters.

scDCCA[30]: integrates dual-contrast learning and denoising encoders to facilitate the model in learning cluster-friendly features at both the instance and cluster levels. This approach enables the acquisition of precise data representations, resulting in improved cell separation.

scGAE[31]: uses a multi-task graph autoencoder to embed structural and feature information, solving the issue of preserving the topological structure of cells during dimensionality reduction.

scGAC[32]: utilizes a graph attention encoder to learn cell embeddings, aggregating neighbor features with varying weights. It employs self-optimizing methods to achieve clustering results.

SCEA[33]: adopts two independent modules (MLP-based encoder and GAT) to learn scRNAseq data and obtain potential representations containing cellular relationships.

Supplementary Section 6: Visualization with predicted and true results

To show the clustering results more intuitive and validate the effectiveness of our scCFIB. We use t-SNE[34] to visualize the results of clusters and true labels on three datasets. Fig. S3 are t-SNE plots with each cell colored by scCFIB predicted clusters and true labels on three different datasets, respectively. We can observe that the plots of each dataset are highly consistent according to the distribution of cell types, which means our scCFIB can accurately separate different cell types from scRNA-Seq data.

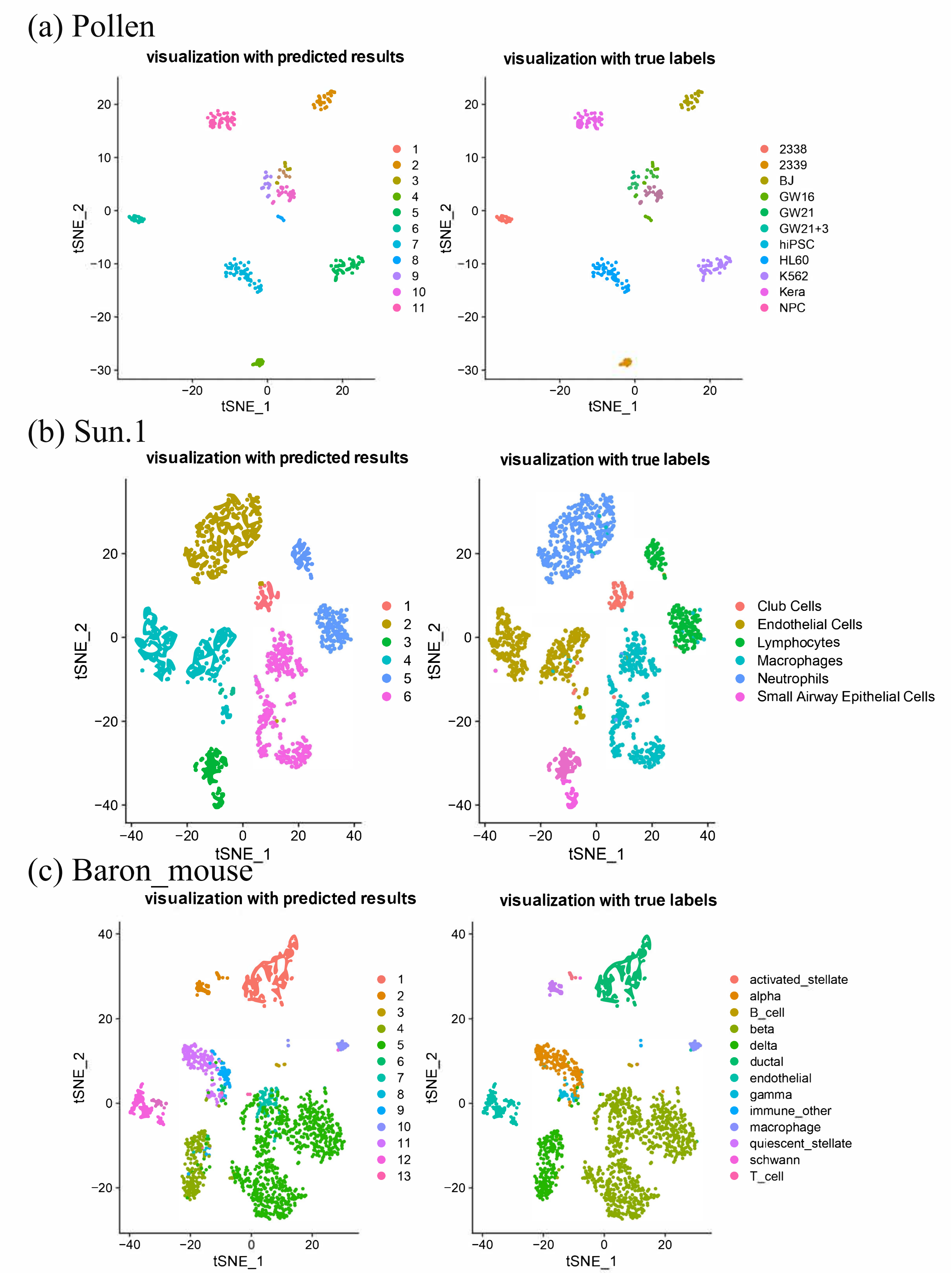

. Figure S3. t-SNE plots of cells are labeled with scCFIB predicted results (left) and true labels (right) on different datasets. (a) The t-SNE plots of the Pollen dataset. (b) The t-SNE plots of the Sun.1 dataset. (c) The t-SNE plots of Baron_mouse dataset.

References

1. Fernando H Biase, Xiaoyi Cao, and Sheng Zhong. Cell fate inclination within 2-cell and 4-cell mouse embryos revealed by single-cell rna sequencing. *Genome research*, 24(11):1787–1796, 2014.
2. Barbara Treutlein, Qian Yi Lee, J Gray Camp, Moritz Mall, Winston Koh, Seyed Ali Mohammad Shariati, Sopheak Sim, Norma F Neff, Jan M Skotheim, Marius Wernig, et al. Dissecting direct reprogramming from fibroblast to neuron using single-cell rna-seq. *Nature*, 534(7607):391–395, 2016.
3. Liying Yan, Mingyu Yang, Hongshan Guo, Lu Yang, Jun Wu, Rong Li, Ping Liu, Ying Lian, Xiaoying Zheng, Jie Yan, et al. Single-cell rna-seq profiling of human preimplantation embryos and embryonic stem cells. *Nature structural & molecular biology*, 20(9):1131–1139, 2013.
4. Mubeen Goolam, Antonio Scialdone, Sarah JL Graham, Iain C Macaulay, Agnieszka Jedrusik, Anna Hupalowska, Thierry Voet, John C Marioni, and Magdalena Zernicka-Goetz. Heterogeneity in oct4 and sox2 targets biases cell fate in 4-cell mouse embryos. *Cell*, 165(1):61–74, 2016.
5. Qiaolin Deng, Daniel Ramsköld, Björn Reinius, and Rickard Sandberg. Single-cell rna-seq reveals dynamic, random monoallelic gene expression in mammalian cells. *Science*, 343(6167):193–196, 2014.
6. Florian Buettner, Kedar N Natarajan, F Paolo Casale, Valentina Proserpio, Antonio Scialdone, Fabian J Theis, Sarah A Teichmann, John C Marioni, and Oliver Stegle. Computational analysis of cell-to-cell heterogeneity in single-cell rna-sequencing data reveals hidden subpopulations of cells. *Nature biotechnology*, 33(2):155–160, 2015.
7. Alex A Pollen, Tomasz J Nowakowski, Joe Shuga, Xiaohui Wang, Anne A Leyrat, Jan H Lui, Nianzhen Li, Lukasz Szpankowski, Brian Fowler, Peilin Chen, et al. Low-coverage single-cell mrna sequencing reveals cellular heterogeneity and activated signaling pathways in developing cerebral cortex. *Nature biotechnology*, 32(10):1053–1058, 2014.
8. Spyros Darmanis, Steven A Sloan, Ye Zhang, Martin Enge, Christine Caneda, Lawrence M Shuer, Melanie G Hayden Gephart, Ben A Barres, and Stephen R Quake. A survey of human brain transcriptome diversity at the single cell level. *Proceedings of the National Academy of Sciences*, 112(23):7285–7290, 2015.
9. Woosung Chung, Hye Hyeon Eum, Hae-Ock Lee, Kyung-Min Lee, Han-Byoel Lee, Kyu-Tae Kim, Han Suk Ryu, Sangmin Kim, Jeong Eon Lee, Yeon Hee Park, et al. Single-cell rna-seq enables comprehensive tumour and immune cell profiling in primary breast cancer. *Nature communications*, 8(1):15081, 2017.
10. Aleksandra A Kolodziejczyk, Jong Kyoung Kim, Jason CH Tsang, Tomislav Ilicic, Johan Henriksson, Kedar N Natarajan, Alex C Tuck, Xuefei Gao, Marc Bühler, Pentao Liu, et al. Single cell rna-sequencing of pluripotent states unlocks modular transcriptional variation. *Cell stem cell*, 17(4):471–485, 2015.
11. Zhe Sun, Li Chen, Hongyi Xin, Yale Jiang, Qianhui Huang, Anthony R Cillo, Tracy Tabib, Jay K Kolls, Tullia C Bruno, Robert Lafyatis, et al. A bayesian mixture model for clustering droplet-based single-cell transcriptomic data from population studies. *Nature communications*, 10(1):1649, 2019.
12. Maayan Baron, Adrian Veres, Samuel L Wolock, Aubrey L Faust, Renaud Gaujoux, Amedeo Vetere, Jennifer Hyoje Ryu, Bridget K Wagner, Shai S Shen-Orr, Allon M Klein, et al. A single-cell transcriptomic map of the human and mouse pancreas reveals inter-and intra-cell population structure. *Cell systems*, 3(4):346–360, 2016.
13. Mauro J Muraro, Gitanjali Dharmadhikari, Dominic Grün, Nathalie Groen, Tim Dielen, Erik Jansen, Leon Van Gurp, Marten A Engelse, Francoise Carlotti, Eelco Jp De Koning, et al. A single-cell transcriptome atlas of the human pancreas. *Cell systems*, 3(4):385–394, 2016.
14. Allon M Klein, Linas Mazutis, Ilke Akartuna, Naren Tallapragada, Adrian Veres, Victor Li, Leonid Peshkin, David A Weitz, and Marc W Kirschner. Droplet barcoding for single-cell transcriptomics applied to embryonic stem cells. *Cell*, 161(5):1187–1201, 2015.
15. Roman A Romanov, Amit Zeisel, Joanne Bakker, Fatima Girach, Arash Hellysaz, Raju Tomer, Alan Alpar, Jan Mulder, Frederic Clotman, Erik Keimpema, et al. Molecular interrogation of hypothalamic organization reveals distinct dopamine neuronal subtypes. *Nature neuroscience*, 20(2):176–188, 2017.
16. Grace XY Zheng, Jessica M Terry, Phillip Belgrader, Paul Ryvkin, Zachary W Bent, Ryan Wilson, Solongo B Ziraldo, Tobias D Wheeler, Geoff P McDermott, Junjie Zhu, et al. Massively parallel digital transcriptional profiling of single cells. *Nature communications*, 8(1):14049, 2017.
17. Chrysothemis C Brown, Herman Gudjonson, Yuri Pritykin, Deeksha Deep, Vincent-Philippe Lavallée, Alejandra Mendoza, Rachel Fromme, Linas Mazutis, Charlotte Ariyan, Christina Leslie, et al. Transcriptional basis of mouse and human dendritic cell heterogeneity. *Cell*, 179(4):846–863, 2019.
18. Xiaoping Han, Renying Wang, Yincong Zhou, Lijiang Fei, Huiyu Sun, Shujing Lai, Assieh Saadatpour, Ziming Zhou, Haide Chen, Fang Ye, et al. Mapping the mouse cell atlas by microwell-seq. *Cell*, 172(5):1091–1107, 2018.
19. Kun Yin, Meijuan Zhao, Li Lin, Yingwen Chen, Shanqing Huang, Chun Zhu, Xuan Liang, Fanghe Lin, Haopai Wei, Huimin Zeng, et al. Well-paired-seq: a size-exclusion and locally quasi-static hydrodynamic microwell chip for single-cell rna-seq. *Small Methods*, 6(7):2200341, 2022.
20. Malte D Luecken and Fabian J Theis. Current best practices in single-cell rna-seq analysis: a tutorial. *Molecular systems biology*, 15(6):e8746, 2019.
21. L Hubert and P Arabie. Comparing partitions journal of classification 2 193–218. *Google Scholar*, pages 193–128, 1985.
22. Alexander Strehl and Joydeep Ghosh. Cluster ensembles—a knowledge reuse framework for combining multiple partitions. *Journal of machine learning research*, 3(Dec):583–617, 2002.
23. Nguyen Xuan Vinh and Julien Epps. Bailey, j2738784: Information theoretic measures for clusterings comparison: variants, properties, normalization and correction for chance. vol. 11. *J Mach Learn Res*, 11:2837–2854, 2010.

24. Edward B Fowlkes and Colin L Mallows. A method for comparing two hierarchical clusterings. *Journal of the American statistical association*, 78(383):553–569, 1983.
25. Naftali Tishby, Fernando C Pereira, and William Bialek. The information bottleneck method. *arXiv preprint physics/0004057*, 2000.
26. Vladimir Yu Kiselev, Kristina Kirschner, Michael T Schaub, Tallulah Andrews, Andrew Yiu, Tamir Chandra, Kedar N Natarajan, Wolf Reik, Mauricio Barahona, Anthony R Green, et al. Sc3: consensus clustering of single-cell rna-seq data. *Nature methods*, 14(5):483–486, 2017.
27. Bo Wang, Daniele Ramazzotti, Luca De Sano, Junjie Zhu, Emma Pierson, and Serafim Batzoglou. Simlr: A tool for large-scale genomic analyses by multi-kernel learning. *Proteomics*, 18(2):1700232, 2018.
28. Tian Tian, Ji Wan, Qi Song, and Zhi Wei. Clustering single-cell rna-seq data with a model-based deep learning approach. *Nature Machine Intelligence*, 1(4):191–198, 2019.
29. Xinwei He, Kun Qian, Ziqian Wang, Shirou Zeng, Hongwei Li, and Wei Vivian Li. scace: an adaptive embedding and clustering method for single-cell gene expression data. *Bioinformatics*, 39(9):btad546, 2023.
30. Jing Wang, Junfeng Xia, Haiyun Wang, Yansen Su, and Chun-Hou Zheng. scdcca: deep contrastive clustering for single-cell rna-seq data based on auto-encoder network. *Briefings in Bioinformatics*, 24(1):bbac625, 2023.
31. Zixiang Luo, Chenyu Xu, Zhen Zhang, and Wenfei Jin. A topology-preserving dimensionality reduction method for single-cell rna-seq data using graph autoencoder. *Scientific reports*, 11(1):20028, 2021.
32. Yi Cheng and Xiuli Ma. scgac: a graph attentional architecture for clustering single-cell rna-seq data. *Bioinformatics*, 38(8):2187–2193, 2022.
33. Saeedeh Akbari Rokn Abadi, Seyed Pouria Laghaee, and Somayyeh Koohi. An optimized graph-based structure for single-cell rna-seq cell-type classification based on non-linear dimension reduction. *BMC genomics*, 24(1):227, 2023.
34. G Hinton and L Van Der Maaten. Visualizing data using t-sne journal of machine learning research. *Journal of Machine Learning Research*, 9:2579–2605, 2008.
